# Social Media and eHealth Literacy Among Older Adults: Systematic Literature Review

**DOI:** 10.2196/66058

**Published:** 2025-03-26

**Authors:** Chenglin Zhang, Emma Mohamad, Arina Anis Azlan, Anqi Wu, Yilian Ma, Yihan Qi

**Affiliations:** 1 Centre for Research in Media and Communication Faculty of Social Sciences and Humanities Universiti Kebangsaan Malaysia Selangor Malaysia; 2 Komunikasi Kesihatan (Healthcomm)-Universiti Kebangsaan Malaysia Research Group Universiti Kebangsaan Malaysia Selangor Malaysia

**Keywords:** eHealth literacy, digital health literacy, older adults, social media, health information, systematic review

## Abstract

**Background:**

The advent of social media has significantly transformed health communication and the health-related actions of older adults, offering both obstacles and prospects for this generation to embrace eHealth developments.

**Objective:**

We aimed to investigate the correlation between social media and eHealth literacy in older individuals and answer four research questions: (1) What are the specific social media behaviors (including general use behaviors and health behaviors) of older adults on social media? (2) How do these behaviors impact their eHealth literacy? (3) How does eHealth literacy influence older adults’ social media behaviors? and (4) What factors influence older adults’ use of social media for health-related purposes?

**Methods:**

Using predetermined keywords and inclusion criteria, we searched Scopus, Web of Science, and PubMed databases for English-language journal articles published from 2000 to 2024, following the PRISMA (Preferred Reporting Items for Systematic Reviews and Meta-Analyses) principles. The initial search identified 1591 publications, and after removing duplicates, 48.21% (767/1591) of publications remained. Ultimately, 1% (16/1591) of studies met the inclusion criteria. A research question–driven manual qualitative thematic analysis was conducted, guided by the categorization of social media use behaviors, the definition of eHealth literacy, and the social-ecological model to provide direction for coding and thematic analysis. In addition, attention was given to identifying unanticipated behaviors or phenomena during the coding process, and these were subsequently incorporated into the analytical framework.

**Results:**

The results indicated that older adults’ general social media use behaviors are primarily characterized by social media preferences, with 2 subthemes identified. Their social media health behaviors revealed 5 main themes and 14 subthemes. Among the primary themes, health information behavior appeared most frequently (12/16, 75%), followed by self-management (8/16, 50%). Other themes included health decision-making (4/16, 25%), telemedicine (3/16, 19%), and health interventions (2/16, 13%). Cross-thematic analysis confirmed that older adults’ social media use behaviors and their eHealth literacy had a reciprocal relationship. Finally, the study revealed that the use of social media to improve eHealth literacy among older adults was influenced by individual, interpersonal, institutional or organizational, and social factors.

**Conclusions:**

The reciprocal relationship between older adults’ social media use and eHealth literacy highlights the importance of establishing a long-term positive mechanism that mutually reinforces social media health practices and eHealth literacy. Based on the findings, this review proposes key directions for efforts to achieve this goal: (1) leveraging postpandemic momentum to enhance eHealth literacy among older adults through social media, (2) reconsidering the dimensions of eHealth literacy among older adults in the context of Web 2.0, (3) actively developing age-friendly integrated social media health service platforms, (4) optimizing social media for engaging and reliable health information for older adults, and (5) integrating social support systems to foster lifelong eHealth learning for older adults.

## Introduction

### Background

The continuous proliferation and extensive use of social media have deeply revolutionized the dissemination of information, transformed the health care industry, and advanced the progress of eHealth. Social media platforms refer to online platforms that enable individuals to engage in interactions and present themselves selectively, either in real time or with a delay, to both wide and specific audiences [1], which have had robust expansion in recent years. Presently, the global monthly active users of the 2 prominent social media platforms, Facebook and Twitter, have attained sums of 3.06 billion and 2.5 million accordingly [2]. Especially during the COVID-19 pandemic, the adoption of social media in health areas was maximized. In the presence of lockdown and social distancing measures, social media emerged as the most effective platform for individuals to obtain health-related information [3,4]. The introduction of social media signaled a fundamental change in the field of eHealth communication. Its widespread use and robustness have established social media as a potential platform for the public to obtain health information [5,6], improve their health behaviors [7], promote social support [8-10], manage diseases, and choose health care institutions [11].

For older adults, who are often marginalized in digital innovation, social media offers a potential solution to bridge the health communication gap and promote active aging. According to the Pew Internet report [12] released in 2021, older adults are among the fastest-growing groups using social media. Moreover, social media use among older adults has grown further with the outbreak of the COVID-19 pandemic [13]. With the increasing use of social media among older adults, their opportunities to engage in eHealth are also expanding. Unlike younger individuals, who prefer a variety of software options, older adults tend to favor a single platform [14]. Therefore, compared to other specialized digital health platforms, social media serves as a more familiar and user-friendly integrated platform for health information, social support, and health care services for older adults. This effectively lowers the barriers for older adults to adopt eHealth technologies. Recently, some research has indicated that social media can effectively enable older individuals to access health information and knowledge, thereby improving their health management [15,16]. Moreover, the experience of social media has been found to be associated with older adults’ willingness to adopt telemedicine [17].

However, it does not mean that there is no threshold for older adults to truly benefit from eHealth technology via social media. eHealth literacy, which is a crucial determinant of an individual’s use of health technology [18], is an essential competency that older adults need to possess to successfully integrate into the eHealth trend. The notion of eHealth literacy, initially introduced by Norman and Skinner [19], refers to the “ability to seek, find, understand, and appraise health information from electronic sources and apply the knowledge gained to addressing or solving a health problem.” However, the definition was presented in the context of Web 1.0, ignoring the internet-based nature of technology in Web 2.0. The definition has subsequently been updated by several scholars [20-26]. The interactivity of eHealth systems is an important element that most scholars focus on in the new definition. In particular, Paige et al [26] developed the concept and content of eHealth literacy based on the transactional model of communication, further emphasizing the interactivity factor. They define eHealth literacy as “...the ability to locate, understand, exchange, and evaluate health information from online environments in the presence of dynamic contextual factors and to apply the knowledge gained across ecological levels for the purposes of maintaining or improving health” [26]. Their conceptualization not only preserves the fundamental elements of conventional eHealth literacy but also underscores that *communication* refers to the capacity to establish connections and identities with other internet users by exchanging health-related information, which sheds light on our understanding of eHealth literacy based on the setting of Web 2.0.

eHealth literacy profoundly influences the effectiveness of an individual’s use of social media to engage in eHealth. Because the credibility of information on social media is lower than that of professional websites, eHealth literacy, particularly critical health literacy, is essential to help evaluate the reliability of the information [27,28]. In addition, high eHealth literacy enables individuals to be better informed and equipped to access, share, and discuss health-related information and issues [29] and gain better satisfaction with the health information [30]. Therefore, eHealth literacy is a crucial skill that older adults must possess to successfully integrate into the eHealth trend through social media.

However, a harsh reality is that eHealth literacy among older people is generally found to be low compared to the younger generation [31-33]. Research has found that lower eHealth literacy makes it challenging for older adults to participate in eHealth, widening the digital divide in eHealth [34-36]. Older adults are perceived to be targets of misinformation and fraud on social media platforms and are more vulnerable to online scams [37]. They are very concerned about their privacy and security when sharing information on social media, and they consider this conduct to be highly risky [37,38]. Moreover, older adults tend to lack the perceived usefulness and ease of use of new technologies, making them slower to adopt these technologies compared to younger individuals [39], and thus they are more likely to encounter technical difficulties. These challenges not only prevent older people from truly benefiting from eHealth but may also lead to a digital divide in eHealth.

Another trend worth noting is the significant potential of social media itself to enhance eHealth literacy. Previous studies have found a positive correlation among individuals’ proficiency in using social media, their level of activity, and the number of social media platforms they use with their eHealth literacy [9,10]. In particular, the frequent use of social media to search for and engage with health information holds great potential for improving individual eHealth literacy [40]. Furthermore, the social support provided by social media can effectively enhance individuals’ understanding of health information and their ability to make informed health decisions, thereby improving eHealth literacy [9]. Social media is also an essential tool for eHealth literacy interventions. For example, Jiang [41] found that health interventions implemented through community-based, proactive health management apps on social media not only increased patients’ engagement in health management but also improved their eHealth literacy. Similar findings have been observed in studies involving older adults. Research indicates a significant association between older adults’ health-related behaviors on social media and their eHealth literacy levels [42]. A notable characteristic is the heightened use of social media and enhanced eHealth awareness among older individuals following the COVID-19 pandemic [13,43,44].

Therefore, within the context of Web 2.0, a highly promising approach to effectively integrating older adults into the eHealth landscape is to establish a virtuous cycle where their effective use of social media for health-related behaviors reinforces their eHealth literacy. On the one hand, older adults can use familiar social media platforms to access health information and engage in health management or health interventions, gradually enhancing their eHealth literacy through practice. On the other hand, as their eHealth literacy improves, their ability to effectively use social media for eHealth engagement and their satisfaction with these interactions will also increase, ultimately bridging the digital divide in the eHealth domain.

To achieve this goal, it becomes urgent to explore the relationship between social media and eHealth literacy among older adults. Understanding older adults’ social media use patterns—such as frequency of use and platform preferences—along with the impact of health information–seeking behaviors, health management, and health interventions conducted via social media on their eHealth literacy, is essential for identifying both the potential and barriers of social media in promoting health and enhancing eHealth literacy for this population. Simultaneously, examining the influence of eHealth literacy on older adults’ ability to effectively use social media for health-related engagement can help identify current gaps in their eHealth literacy and propose targeted interventions. By deeply grasping the relationship between social media and eHealth literacy, older adults can comprehensively and systematically build a social media–based eHealth integration platform that meets their level of eHealth literacy. Ultimately, a virtuous cycle of mutual promotion between social media and eHealth literacy of older individuals will be formed.

Existing systematic literature studies on eHealth literacy in older individuals have mostly concentrated on examining the determinants that impact eHealth literacy in this population [45] or the intervention [32,46]. Some studies on social media and older adults’ health primarily focus on the impact of social media on their mental health [47,48]. However, few studies systematically and comprehensively review the behaviors of older adults using social media for health purposes, nor do they summarize the correlations between these behaviors and health outcomes. In particular, research on behavioral patterns and preferences of older adults engaging with health-related content on social media; the role of platform design in promoting health literacy; how older adults’ eHealth literacy influences their health-related behaviors on social media; and how these behaviors, in turn, affect their eHealth literacy is limited. Without a clear understanding of these dynamics, the significance and potential of social media as a tool for enhancing older adults’ eHealth literacy and bridging the digital divide in eHealth remain insufficiently elucidated. This gap may ultimately turn social media from a promising tool for improving older adults’ health into a potential weapon that harms this vulnerable population. Furthermore, with the outbreak and end of the COVID-19 pandemic, the user landscape of social media and the developmental landscape of eHealth technology have both changed, necessitating a reassessment of social media use patterns and older individuals’ eHealth literacy.

### Research Questions

This systematic literature review addressed these gaps by focusing on the correlation between older adults’ social media behavior, including general social media use behavior (eg, social media use patterns or habits) and social media health behaviors (eg, health information behaviors, self-management, or interventions), and their level of eHealth literacy. Specifically, this study aimed to answer the following research questions (RQs):

RQ1: What are the specific social media behaviors (including general use behavior and health behaviors) of older adults on social media?

RQ2: How do these behaviors impact their eHealth literacy?

RQ3: How does eHealth literacy influence older adult’s social media behaviors?

RQ4: What factors influence older adults’ use of social media for health-related purposes?

## Methods

### Overview

Through a comprehensive literature review following the PRISMA (Preferred Reporting Items for Systematic Reviews and Meta-Analyses) criteria [49], this review investigated the relationship between social media and older individuals’ eHealth literacy and associated characteristics. PRISMA checklist is provided in Multimedia Appendix 1. We did not register the procedure for this review to a systematic review database.

### Data Resources and Search Strategy

Scopus, Web of Science, and PubMed were selected as databases for this review. The online database search was conducted on May 17, 2024. Although the notion of eHealth literacy was initially presented in 2006 [19], there is some previous literature that may use other vocabularies, such as computer health literacy and internet health literacy, to refer to eHealth literacy. To ensure the thoroughness of the assessment, the literature search was conducted within the period framework of 2000 to 2024. Search terms were generated around the following research themes: “social media,” “eHealth literacy,” and “older people.” Because eHealth literacy is a broad concept, this study not only covers the synonyms of eHealth literacy but also expands on the definition provided by Norman [19] and Paige [26] by adding related content about what eHealth literacy encompasses, such as health literacy, digital literacy, information literacy, and media literacy. Furthermore, the objective of this study was to investigate the correlation between social media and the eHealth literacy of older adults, as well as associated elements. Consequently, the primary focus was given to social networking platforms that facilitate communication and interaction with the public regarding health matters, health information, and health management. Therefore, the definition provided by Carr and Hayes [1] was adopted, and the latest global user activity rankings were used to filter the top-ranked social media as search extensions [2]. All keywords were searched in the database in combination with Boolean logic. A comprehensive list of search queries is presented in Textbox 1. A separate search string for each database is provided in Multimedia Appendix 2.

**Keywords search strings (the asterisk is a wildcard used to include multiple word variations in the search query).**

**Older adults**

- “old*” OR “elder*” OR “senior*” OR “aging*” OR “ageing*” OR “aged*” OR “geriatric*” OR “gerontolog*” OR “retiree*” OR “baby boomer” OR “pensioner*” OR “silver generation”

AND

**eHealth literacy**

- “eHealth literacy” OR “eHealth literacy” OR “telehealth literacy” OR “mHealth literacy” OR “health literacy” OR “medic* literacy” OR “digital literacy” OR “information literacy” OR “media literacy” OR “internet literacy” OR “computer literacy” OR “virtual literacy” OR “intelligent literacy” OR “technolog* literacy” OR “online literacy” OR “web based literacy” OR “Web 2.0 literacy” OR “ICT literacy” OR “Information and Communication Technology literacy”

AND

**Social media**

- “social media” OR “social network*” OR “social platform*” OR “social web*” OR “web 2.0” OR “online social*” OR “digital social*” OR “virtual social*” OR “social channels” OR “social apps” OR “community media*” OR “community platform” OR Facebook OR “YouTube” OR WhatsApp OR Instagram OR WeChat OR “TikTok” OR Douyin OR Telegram OR Snapchat OR Kuaishou OR “Sina Weibo” OR QQ OR Twitter OR Pinterest

### Study Selection Criteria

The selected papers for review had to adhere to the pre-established inclusion criteria. First, the included papers should have been primary research articles written in English and published between January 2000 and May 2024. Other document types, such as reviews, editorials, conference papers, letters, notes, commentaries, book chapters, books, or study protocols, were excluded from the considered literature. Second, the participants under study must have been older individuals. Given the absence of a universally defined age for older individuals, the United Nations generally designates individuals aged ≥60 years as older individuals [50]. However, some articles use national retirement ages as criteria for the research population [42] or define older people as those aged ≥50 years from the perspective of cognitive aging [51]. Considering the diversity in defining older people and the current minimum retirement age of 50 worldwide [52], this review included participants aged ≥50 years to ensure a broader spectrum of articles. Furthermore, it is anticipated that the papers will pertain to eHealth literacy among older persons who are active on social media and investigate the elements that impact their use of social media to enhance their eHealth literacy. Considering this criterion, the following articles were omitted: (1) discussions concerning phone calls, SMS text messages, email, web portals, and telemedicine or mobile health (mHealth) without social media engagement; (2) articles that were not based on health contexts, such as internet literacy, media literacy, digital literacy, and information literacy, for older adults; and (3) articles that were based on internet or social media behaviors of older adults unrelated to eHealth literacy. Furthermore, this paper identified a correlation between eHealth literacy and the social media activities of older people. Details on the particular inclusion and exclusion criteria can be found in Textbox 2.

**Study selection criteria.**

**Inclusion criteria**

- Research topic: eHealth literacy and social media use in the older population
- Research content: articles that investigate the influence and associated variables of social media on eHealth literacy in the older population; articles that examine the effect of older individuals’ eHealth literacy on their social media activities
- Target group: various community groups, including marginalized migrant populations, retirees, and those aged ≥50 years
- Article type: primary research journal articles
- Language: English
- Timeline: between 2000 and 2024

**Exclusion criteria**

- Research topic: topics such as internet literacy, media literacy, digital literacy, and information literacy among older adults in non–health-related contexts; health behaviors of older people based on nonsocial platforms, such as phone calls, SMS text messages, email, web portals, and telemedicine or mobile health without social media engagement; internet or social media behaviors of older adults unrelated to eHealth literacy
- Research content: articles in which the correlation between eHealth literacy and social media use among older people was not explicitly referenced
- Target group: children, adolescents, middle-aged group, and other young groups aged <50 years; study population without a clear age range
- Article type: reviews, editorials, conference papers, letters, notes, commentaries, book chapters, books, or study protocols
- Language: non-English
- Timeline: before 2000

### Literature Screening

There were 3 steps in literature screening. The first step was removing duplicates. Articles identified in the database were filtered into Microsoft Excel and duplicates were removed. Following this was the process of screening the title and abstract. A preliminary screening was conducted by 2 reviewers (CZ and AW) who independently identified important themes in the title and abstract. The age range of the study population was first identified, and studies where the population was not aged ≥50 years were excluded. If the age range could not be identified from the title and abstract, the article was reviewed for relevance to the topic, and theme-related studies were included for full-text review; otherwise, they were excluded. For example, the study by Willis [53] was included for full-text review because the abstract mentioned interviews with online health community members without specifying their ages. However, the full-text review revealed that participants were aged 37 to 85 years, resulting in this study’s exclusion.

The last step was full-text screening. Following the initial screening, the selected articles were reviewed in full text. During this process, 2 independent researchers carefully and comprehensively reviewed the full texts of the selected studies. This stage prioritized the exclusion of literature that did not fit the language, type of article, and age of the study population. Among them, the study conducted by Aung et al [54] was omitted from consideration because it was only a research plan.

Subsequent full-text reviews primarily excluded literature that did not address social media or was not explicitly about social media, as well as literature from nonhealth contexts. For example, some (39/153, 25.5%) articles, such as Mao et al [55] and Evans et al [56] discussed the association between telemedicine or mHealth and eHealth literacy in older adults, but these technologies did not involve social media platforms and were, therefore, excluded. Moreover, a few (4/153, 2.6%) articles discussed internet behaviors and eHealth literacy among older individuals, but it was unclear whether internet behavior related to social media was excluded [57-60]. In addition, some (3/153, 2%) articles, while exploring important components of eHealth literacy in older adults, such as digital literacy, information literacy, or media literacy, were not based on a health context and were, therefore, excluded [61-63]. Notably, several (3/153, 2%) papers examined the influence of social media on alleviating loneliness among older individuals, which did not address eHealth literacy and were excluded [64-66]. Finally, some (3/153, 2%) articles, while dealing with social media and eHealth literacy, did not allow the interaction between the 2 to be judged and were, therefore, excluded [67-69].

For articles that met the inclusion criteria in the full-text review, we annotated the study population, the research methodology, and the relevant factors in preparation for subsequent data extraction. To ensure the reliability of the study, a cross-review was conducted, and YM was introduced to resolve any discrepancies.

### Quality Appraisal

This review used the quality assessment (QA) tool developed by Sirriyeh et al [69] to evaluate the quality of the included studies. The QA tool establishes 6 QAs and assigns scores on a 3-point scale from 0 to 1 (0=no, 0.5=partly, and 1=yes). Studies with an overall score of >3 points are recognized as being of sufficient quality. Two researchers (CZ and AW) independently scored the included studies, and the sum of the scores obtained was used to assess the study quality. Inconsistent results were resolved by third-party (YM) intervention.

### Data Extraction and Analysis

To capture the basic information and trends of the studies included, we developed a standardized table summarizing the fundamental characteristics of the research. This table extracted details, such as the authors, publication year, country, participants’ age, and research methods, from the included literature. Furthermore, to clarify the roles played by different types of social media, we extracted and categorized the social media platforms mentioned in the articles. In addition, as no restrictions were imposed on the research methods of the included studies, which covered qualitative, quantitative, and mixed methods approaches, thematic analysis [70] was used to describe and analyze the literature. According to Braun and Clarke [70], thematic analysis is a flexible research tool with a high degree of theoretical freedom, making it applicable within various theoretical frameworks and capable of providing rich and detailed data descriptions.

**Figure 1:**
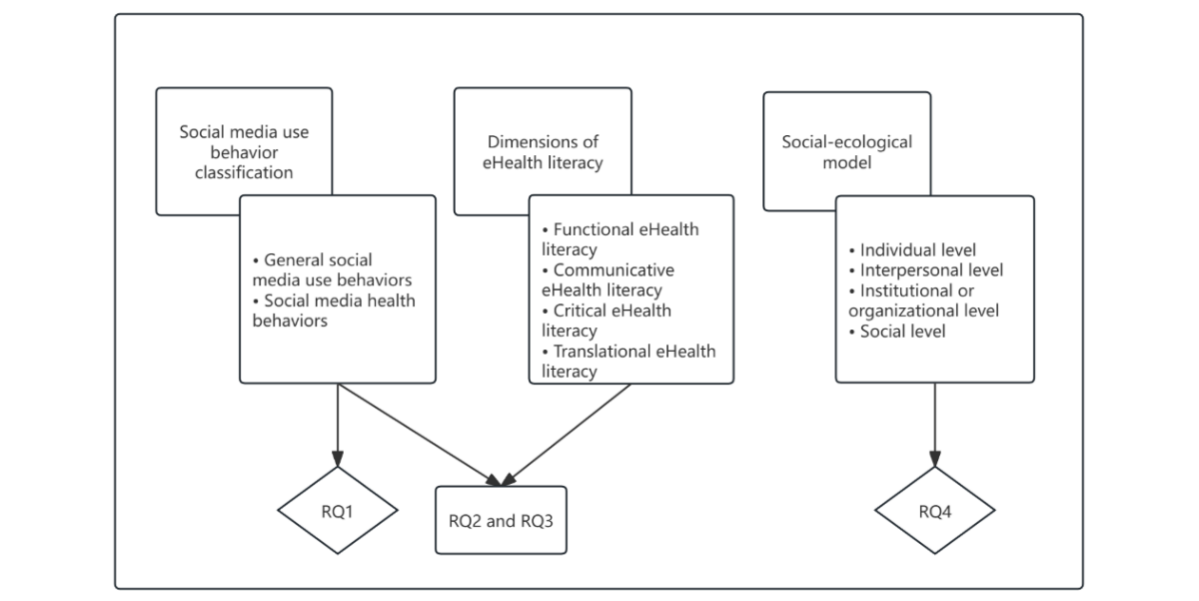
Qualitative thematic analysis framework map. RQ: research question.

We conducted a manual, qualitative content analysis guided by the RQs, integrating classifications of social media health behaviors, definitions of eHealth literacy [26], and the social-ecological model (SEM) [71] to inform the coding and thematic analysis. At the same time, we paid attention to identifying new behaviors or phenomena not initially anticipated and incorporated them into the analytical framework. The qualitative thematic analysis framework is illustrated in Figure 1.

Specifically, guided by RQ1, in addition to focusing on older adults’ general social media behaviors (including use habits, such as frequency of use and platform preferences), we reviewed the literature on the application of social media in the health domain. This allowed us to extract consumer-related behavioral manifestations resulting from the use of social media in health contexts. As both health consumers and professionals can act as providers or recipients of support during interactions on health platforms [72], we examined health behaviors not only from the perspective of consumers or patients but also from the perspectives of health care professionals or other organizations interacting with consumers or patients. Our analysis identified key consumer-related applications of social media in health, including health information (searching, sharing, and evaluating); telemedicine; health management; health education; health promotion; and health intervention [73-76]. These applications served as the foundation for our coding and thematic extraction. To ensure rigor, we used the “conceptual matrix” proposed by Webster and Watson [77] (Multimedia Appendix 3 [13,14,42,44,51,69,78-88]) to examine themes and systematically record emerging themes during the analysis process.

RQ2 and RQ3 explored the correlation between older adults’ health behaviors on social media and their eHealth literacy. We adopted the definition of eHealth literacy provided by Paige et al [26] as the guiding framework for thematic analysis. Paige et al [26] delineated eHealth literacy into four dimensions:

1. Functional eHealth literacy: basic skills in reading and writing (typing) about health to effectively function on the internet
2. Communicative eHealth literacy: the ability to collaborate, adapt, and manage communication about health in social online environments with multimedia
3. Critical eHealth literacy: the ability to evaluate the credibility, relevance, and risks of sharing and receiving health information on the internet
4. Translational eHealth literacy: the ability to apply health knowledge gained from the internet across diverse ecologic contexts

We extracted descriptions from the literature relevant to these 4 dimensions and performed theoretical mapping to determine the eHealth literacy dimensions they pertained to. For studies that only referred to eHealth literacy as a general concept, we assumed that they encompassed all 4 dimensions.

Finally, we conducted a cross-analysis between older adults’ social media use behaviors—including general and health-related behaviors (as identified in the themes extracted based on RQ1)—and the 4 dimensions of eHealth literacy. This analysis aimed to uncover the relationships between the 2, focusing on whether older adults’ social media use behaviors enhance or hinder their eHealth literacy and whether their eHealth literacy strengthens or diminishes their health-related behaviors on social media. Given the diversity of research methods in the included studies, our qualitative content analysis not only focused on explicit conclusions from studies that directly examined these relationships but also considered implicit or related findings within the research.

Guided by RQ4, we examined the factors influencing older adults’ use of social media to access health information or health services in each article. Because these factors included not only barriers and facilitators but also neutral factors, we adopted the SEM to enable a more comprehensive extraction and integration of these influencing factors. SEM, developed by Bronfenbrenner [71], provides a systematic framework for understanding the impact of various environmental elements on individual behavior and development. According to SEM, the influencing factors are categorized into 4 dimensions: individual, interpersonal, institutional or organizational, and social levels.

CZ and AW analyzed all the articles and extracted the relevant themes. AAA and YQ reviewed the full text of the articles to validate the extracted themes. All conflicts were resolved by EM. Finally, multiple meetings were held to discuss the results of the thematic analysis and reach a consensus.

## Results

### Search Results

**Figure 2:**
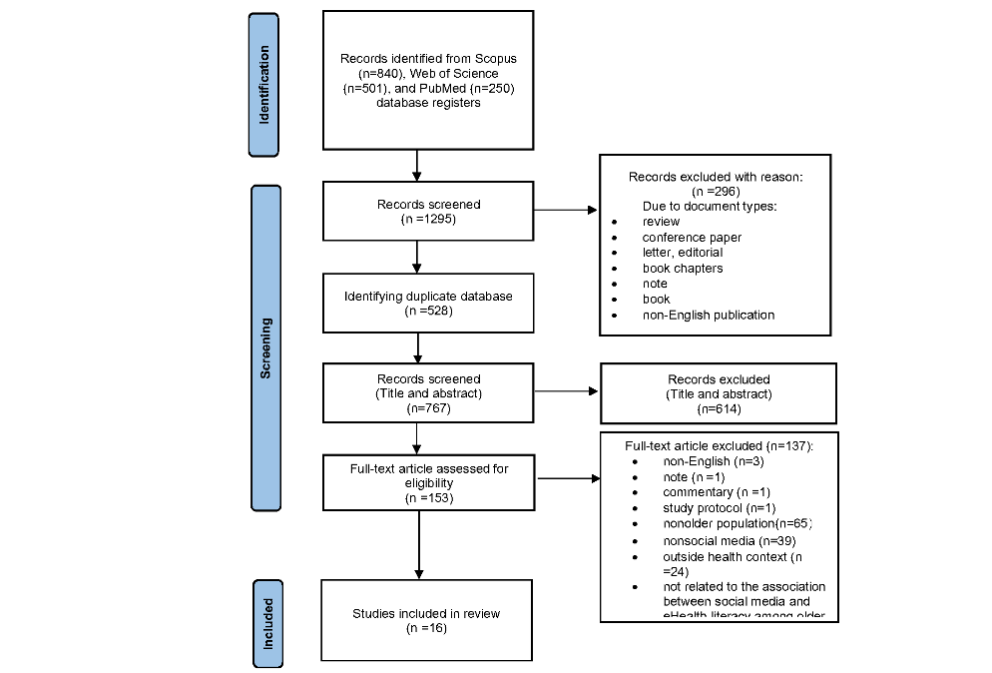
PRISMA (Preferred Reporting Item for Systematic Reviews and Meta-Analyses) flow diagram depicting the process of study selection.

Search results and data collection process are shown in the PRISMA flow diagram in Figure 2. Initially, 1591 articles were identified (Scopus, 840/1591, 52.79%; Web of Science, 501/1591, 31.49%; and PubMed, 250/1591, 15.71%). A total of 48.21% (767/1591) of articles were screened by excluding certain articles based on the exclusion criteria, such as article type, language, and date, and after duplicates were removed. Subsequently, 38.6% (614/1591) of articles were eliminated following the screening of the title and abstract, while 8.6% (137/1591) of articles were eliminated after the assessment of the entire text. Publications were eliminated throughout the full-text screening process for the following reasons: (1) non-English (3/153, 2%), (2) note (1/153, 0.6%), (3) commentary (1/153, 0.7%), (4) study protocol (1/153, 0.7%), (5) younger population (65/153, 42.5%), (6) non–social media (39/153, 25.5%), (7) outside health context (24/153, 15.7%), and (8) not related to the correlation between eHealth literacy and older population (3/153, 2%). Although a considerable amount of research was identified at the outset, many articles used social media only as a tool for the recruitment of research participants or for distributing questionnaires, without discussing social media, and were, therefore, excluded.

### Description of Studies

Comprehensive information regarding the included research is provided in Table 1.

Generally, most (15/16, 94%) articles were published between 2020 and 2024, namely, after the COVID-19 pandemic. Only 1 (6%) study was conducted in 2015 [78]. Nearly two-thirds (11/16, 69%) of the studies were from Asian countries, including China (6/16, 38%) [14,42,44,79-81], Korea (2/16, 13%) [82,83], Thailand (2/16, 13%) [13,84], Singapore (1/16, 6%) [59]. In total, 13% (2/16) of studies were from the United States [85,86], and 1 (6%) study was from Italy [87]. Concerning the age definition of older individuals, except for 2 (13%) papers that did not provide a specific age criterion [87,88], the age definition ranges of the studies mainly used ≥50 years (5/16, 31%) [14,79,80,84,86] or ≥60 years (5/16, 31%) [13,44,80,83,84], and a few studies used ≥65 years (2/16, 13%) [82,85] or ≥55 years (1/16, 6%) [81]. Moreover, 1 (6%) Chinese study used the retirement age as the range of the study population, that is, ≥55 years for women and ≥60 years for men, respectively [42]. Most (12/16, 75%) studies were quantitative (Textbox 2), including surveys (10/16, 62%) [13,42,78-80,82,84,86-88] and experiments (2/16, 13%) [51,83], while 2 (13%) studies used mixed methods [44,85]. Only 2 (13%) studies were qualitative, using interviews [14,81].

**Table 1:** Study characteristics.

Study	Country	Population age (y)	Research method
Matchanova et al [51], 2024	United States	50	Experiment
Ye [80], 2024	China	>60	Survey
Oh et al [86], 2023	United States	>50	Survey
Vitolo et al [87], 2023	Italy	66 to 84	Survey
Jang et al [82], 2023	Korea	>65	Survey
Kachentawa [13], 2023	Thailand	>60	Survey
Lee and Ryu [83], 2023	Korea	>60	Experiment
Wang and Zhang [42], 2023	China	Men: >60 Women: >55	Survey
Liu et al [44], 2022	China	>60	Mixed methods (survey and interview)
Chai [81], 2022	China	>55	Interview
Tan et al [88], 2022	Singapore	56 to 75	Survey
Chen et al [85], 2021	United States	>65	Mixed methods (experiment and interview)
Wu and Yu [14], 2021	China	>50	Interview
Ubolwan et al [84], 2020	Thailand	>60	Survey
Shang and Zuo [79], 2020	China	>50	Survey
Tennant et al [78], 2015	United States	>50	Survey

Regarding features related to social media, most (11/16, 69%) articles discussed a wide range of social media platforms [13,44,78-82,84,86-88], and a few (5/16, 31%) articles explored the role of individual social media only [14,42,51,83,85], covering WeChat (2/16, 13%) [83], Facebook (1/16, 6%) [51], The BAND app (NAVER Corp) (1/16, 6%) [83], and Virtual Online Communities for Aging-Life Experiences (1/16, 6%) [85]. Moreover, social media was mostly (11/16, 69%) regarded as both a medium for health information and health-related relationships [13,42,44,51,78,79,81,82,84,86,88]. Furthermore, 1 (6%) study examined the dual role of social media as a medium for health-related interactions and information [80]. Social media was discussed as a health intervention or health education tool (2/16, 13%) [83,85], and a health management and communication tool (1/16, 6%) [14]. Only 1 (6%) article did not discuss the role of social media [87].

To further capture the characteristics of older adults in using social media platforms, this review extracted and categorized the social media platforms mentioned in the studies included (Table 2). Kordzadeh [72] categorized social media platforms applied in health care into 2 types: online social networks and virtual health communities (VHCs). The former includes large-scale, general-purpose internet platforms, such as Facebook, Twitter, Instagram, and YouTube, which are designed for broad collaboration among internet users. In contrast, the latter consists of social media sites specifically designed for individuals to discuss health-related topics.

On the basis of the included studies, most (11/16, 69%) explored the role of general social media in the health of older adults without specifying the particular platforms. Among the studies that addressed online social network platforms, the most commonly mentioned were WeChat and Facebook. These platforms are widely popular among older adults in their respective regions and are frequently used for accessing and sharing health-related information. Conversely, VHC platforms exhibited a more distinct health-focused orientation. Only 2 (13%) studies in the included literature specifically examined VHC platforms, namely BAND and Virtual Online Communities for Aging-Life Experiences, highlighting their value as tools for online support groups and health interventions [83,85].

**Table 2:** Social media platforms and categorization included in literature (N=16).

Social media	Social media types			Studies		Studies, n (%)
	OSN^a^	VHC^b^				
Unspecified	✓	✓	Ye [80], 2024; Oh et al [86], 2023; Vitolo et al [87], 2023; Jang et al [82], 2023; Kachentawa [13], 2023; Liu et al [44], 2022; Chai [81], 2022; Tan et al [88], 2022; Ubolwan et al [84], 2020; Shang and Zuo et al [79], 2020; and Tennant et al [78], 2015		11 (69)	
WeChat	✓	—^c^	Wang and Zhang [42], 2023 and Wu and Yu [14], 2021		2 (12)	
Facebook	✓	—	Matchanova et al [51], 2024		1 (6)	
BAND	—	✓	Lee and Ryu [83], 2023		1 (6)	
VOCALE^d^	—	✓	Chen et al [85], 2021		1 (6)	

^a^OSN: online social network.

^b^VHC: virtual health community.

^c^Not applicable.

^d^VOCALE: Virtual Online Communities for Aging-Life Experiences.

### Quality of the Included Studies

According to the results of the quality appraisal (Multimedia Appendix 4 [13,14,42,44,51,69,78-88]), the scores of the included studies ranged from 3.5 to 5.5, with all of them exceeding 3, which is considered to be of sufficient quality. Moreover, none of the studies obtained a score of 0 on the individual evaluation items. This suggests that the included studies effectively elucidated the purpose, interest, utility, methodological process, approach concept, comparison and measurement with other similar studies, and limitations of the study on a high level of clarity.

### Social Media Use Behaviors of Older Adults

As this study focused on the relationship between social media and eHealth literacy among older adults, an analysis of their social media use behaviors was conducted. This thematic analysis included both the general use behaviors of older adults and their health-related behaviors stemming from social media use. Table 3 presents the results of the thematic analysis, identifying 2 themes related to general social media use behaviors and 5 themes associated with health-related behaviors.

**Table 3:** Social media use behaviors of older adults (N=16).

Theme, description, and subtheme				Sample quotes		Studies		Studies, n (%)
**Social media use habit**								
	**The frequency of older adults’ social media use, their platform preferences, and so on**							
		Frequency of social media use	“Frail and pre-frail patients used digital tools less frequently and accessed the Internet less frequently compared to robust patients.” [87]		Vitolo et al [87], 2023; Ubolwan et al [84], 2020; and Tennant et al [78], 2015		3 (19)	
		Social media platform preference	“The most widely adopted function for senior citizens was instant messaging... Compared with the diversity of software applications that the younger generation use, older adults were more interested in a limited range of software affordance which benefited them most.” [14]		Wu and Yu [14], 2021		1 (6)	
**Health information**								
	**The fundamental behaviors of older adults related to health information on social media, including searching, sharing, learning, and evaluating health information**							
		Health information acquisition	“...participants obtained information about COVID19 and general health from various resources....” [44]		Ye [80], 2024; Oh et al [86], 2023; Jang et al [82], 2023; Kachentawa [13], 2023; Wang and Zhang [42], 2023; Liu et al [44], 2022; Chai [81], 2022; Tan et al [88], 2022; and Tennant et al [78], 2015		9 (56)	
		Health information sharing	“Among older adults, a higher likelihood of sharing false COVID-19 headlines was associated with lower verbal IQ and numeracy skills.” [51]		Ye [80], 2024; Matchanova et al [51], 2023； Kachentawa [13], 2023; Lee and Ryu [83], 2023; and Wang and Zhang [42], 2023		5 (31)	
		Health information evaluation	“...interviewees were able to evaluate pandemic rumors as false information, but they may not understand the underpinning scientific logic.” [81]		Matchanova et al [51], 2023; Liu et al [44], 2022; Chai [81], 2022; Tan et al [88], 2022; and Tennant et al [78], 2015		5 (31)	
		Health knowledge learning	“Abundant health knowledge resources are available on social media to facilitate technology-enhanced knowledge learning among older adults.” [79]		Shang and Zuo [79], 2020		1 (6)	
		Health information resource trust	“...trust in formal sources of information on its own positively predicts vaccination status...” [88]		Tan et al [88], 2022		1 (6)	
		Frequency of health information use	“...the frequency of using online health information also has a positive influence on the three levels of e-health literacy of older adults.” [80]		Ye [80], 2024		1 (6)	
**Self-management**								
	**Older adults are engaged in social media–based behaviors to manage symptoms, treatments, and lifestyles**							
		Health prevention	“All the older adults consciously followed the prevention and control strategies they had learned...” [44]		Jang et al [82], 2023； Kachentawa [13], 2023； Chai [81], 2022； Liu et al [44], 2022; Chen et al [85], 2021； and Wu and Yu [14], 2021		6 (38)	
		Lifestyle management	“In this study, participants expressed greater appreciation of physical activity and the need to be more proactive about self-management, which could lead to a healthier lifestyle.” [85]		Ye [80], 2024; Chen et al [85], 2021; Chai [81], 2022; and Liu et al [44], 2022		4 (25)	
		Medical management	“During the lockdown, citizens managed their living needs and medical requirements by the ‘grid managed’ online community facilitated by SNS chat groups...” [14]		Wu and Yu [14], 2021 and Oh et al [86], 2023		2 (12)	
**Telemedicine**								
	**Older adults access health care through social media**							
		Communicating with health care providers	“Among the health communication factors, social media use, face-to-face PPC, electronic PPC, and use of electronic communication through electronic patient platforms were facilitating communication factors of CRC screening among NHWs.” [86]		Oh et al [86], 2023		1 (6)	
		Access to health care	“A face-to-face visit with health care providers was limited due to home quarantine and isolation, so older adults also learned to seek medical care through the Internet.” [44]		Liu et al [44], 2022 and Wu and Yu [14], 2021		2 (12)	
**Health decision-making**								
	**Older adults integrate relevant information from social media with their personal knowledge and beliefs to make health decisions that meet their needs**							
		Medical decision	“...those who placed greater trust in social media being less likely to have received at least 1 dose of the vaccine.” [88]		Tan et al [88], 2022 and Kachentawa [13], 2023		2 (12)	
		Addressing general health issues	“With regard to problem-solving, participants mentioned effects they experienced, including that the discussion affected participants’ behavior, either in terms of provoking a more active approach to addressing health issues, or considering the use of the skills in their own lives.” [85]		Ye [80], 2024 and Chen et al [85], 2021		2 (12)	
**Health intervention**								
	**Systematic behavioral changes to improve health outcomes among older adults using social media**							
		Online group interventions	“...a gamified walking program, called the MSG walking program (mHealth devices and Social media apps using Gamified walking program), was designed to enable socializing in conjunction with mHealth apps and devices to operate an online walking program for retired older adults during COVID-19.” [83]		Lee and Ryu [83], 2023 and Chen et al [85], 2021		2 (12)	

The general social media use behaviors of older adults describe their use patterns, including frequency of use, platform preferences, and other related habits. Among the included studies, 19% (3/16) [78,84,87] investigated the time spent or frequency of social media use by older adults. Wu and Yu [14] explored platform preferences among older adults, noting their inclination toward instant text messaging features on social media. Unlike younger users, older adults tended to prefer platforms with limited features and familiar interfaces, rather than overly complex or feature-rich social media platforms.

For the thematic analysis of older adults’ health behaviors on social media, we adopted a combined approach of using predefined themes and identifying emergent themes. Drawing from previous research on the application of social media in health communication, certain themes were established in advance. These themes were further refined and expanded during the full-text review stage. Ultimately, 5 primary themes emerged: health information, health interventions, health decision-making, self-management, and telemedicine—all of which aligned with our predefined themes.

However, in the extraction of subthemes, we uncovered more nuanced information. For instance, while the predefined theme of health information behavior initially encompassed information-seeking, sharing, and evaluation behaviors, the coding process revealed additional discussions by researchers on older adults’ learning of health knowledge, trust in health information sources, and frequency of health information use. For example, the study by Tan et al [88] examined how trust in different information sources influenced older adults’ decisions regarding COVID-19 vaccination [88].

Notably, health information behavior was the most frequently discussed theme, appearing in 75% (12/16) of the studies. Within this category, particular emphasis was placed on information-seeking (9/16, 56%), sharing (5/16, 31%), and evaluation behaviors (5/16, 31%). Furthermore, social media information also impacted older adults’ health decision-making. These decisions were not only limited to medical choices, such as vaccination [13,88], but also extended to addressing daily health-related challenges [80,85].

Self-management emerged as the second most frequently discussed theme (8/16, 50%), referring to older adults’ health management behaviors related to their diseases and lifestyles through social media. Among the aspects of self-management, health prevention was the most extensively explored subtheme (5/16, 31%), primarily due to the prevalence of the COVID-19 pandemic as the context in many studies. In addition to health prevention, older adults use social media for managing their lifestyles [81,85] and medical needs [14,86].

Finally, social media also serves as an important platform for older adults to engage in telemedicine and for implementing health interventions targeted at this population. Health interventions refer to planned efforts to modify older adults’ health behaviors via social media to improve their health outcomes. For instance, Lee and Ryu [83] and Chen et al [85] conducted online group health interventions for older adults using VHC platforms, demonstrating the potential of such platforms in facilitating health-related behavioral changes.

### Correlation Between Social Media Use and the eHealth Literacy in Older Individuals

#### Overview

**Figure 3:**
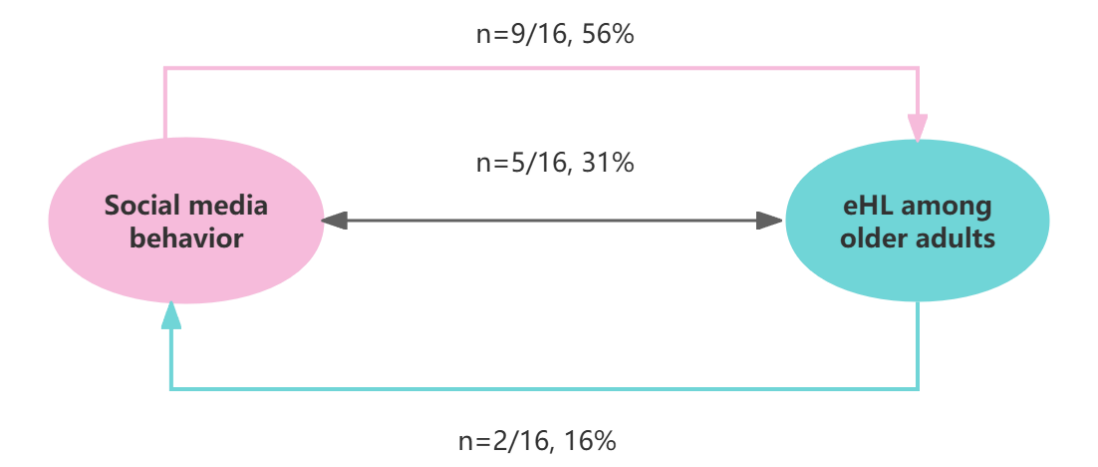
The correlation between social media use and eHealth literacy (eHL) in older individuals. Reference sources for these data include (9/16, 56%) [13,14,78,82-87]; (5/16, 31%) [14,44,80,81,88]; and (2/16, 13%) [42,51].

Figure 3 [13,14,42,44,51,78,80-88] presents the comprehensive correlation between social media use and the eHealth literacy in older adults. The included studies revealed that social media and older adults’ eHealth literacy are mutually influencing and interacting. In total, 56% (9/16) of the articles examined the influence of social media on the eHealth literacy of older individuals [13,14,78,82-87], 13% (2/16) only discussed the value of older adults’ eHealth literacy on their social media use [42,51], and 31% (5/16) covered the interplay between the two [14,44,80,81,88].

#### Impact of Social Media on eHealth Literacy of Older Individuals

##### Overview

The overwhelming majority (13/16, 81%) of the research included confirmed the impact of social media on the eHealth literacy of older individuals (Table 4), and the pathways to impact included social media use habits (4/16, 25%), social media health information behaviors (7/16, 44%), social media–based health interventions (2/16, 13%), telemedicine (2/16, 13%), and health decision-making (1/16, 6%).

Table 5 categorizes and counts the behavioral themes.

**Table 4:** The impact of social media on eHealth literacy (eHL) of older individuals.

Study and social media behavioral dimensions that affect eHL		Theoretical mapping: dimensions of affected eHL	Mechanism of association and key findings
**Ye [80], 2024**			
	Frequency of health information use Health information acquisition Health information sharing Self-management (lifestyle management)	Translational eHL: application ability, decision-making ability Critical eHL: critical judgment ability	The social media health information behavior of older adults (including frequency of use, acquisition, and sharing of health information) and self-management behavior through online health information significantly affect their translational eHL and critical eHL.
**Oh et al [86], 2023**			
	Telemedicine (communicating with health care providers)	Communicative eHL: health communication	Older adults’ use of social media to communicate with health care providers enhances their communicative eHL.
**Vitolo et al [87], 2023**			
	Frequency of social media use	eHL: digital health literacy	A lower frequency of social media use is correlated with lower eHL in older persons.
**Jang et al [82], 2023**			
	Health information acquisition	Translational eHL: application of digital resources and health prevention	Older adults with limited sources of health information (unable to diversify their use of digital information resources, including social media, and traditional resources) exhibit lower digital resource use and health prevention behaviors, hindering their eHL to some extent, especially translational eHL.
**Kachentawa [13], 2023**			
	Health information evaluation Health information sharing Self-management (health prevention) Health decision-making (medical decision)	Critical eHL: understanding and evaluation of health information Functional eHL: ability to learn new things on social media, basic technical skills for using social media, ability to solve basic problems on social media, ability to understand how family explains using social media Translational eHL: ability to apply health information	Literacies related to social media, including media and information literacy, as well as computer and social media literacy (corresponding to critical eHL, functional eHL, and translational eHL), should be included as indicators of older adults’ eHL.
**Lee and Ryu [83], 2023**			
	Health intervention (online group intervention)	eHL	Social media–based, online group health interventions can provide social support for older adults and enhance their eHL.
**Liu et al [44], 2022**			
	Health information acquisition Telemedicine (accessing health care services) Self-management (health prevention)	eHL	During the COVID-19 pandemic, older adults’ use of social media for health information acquisition, telemedicine, and self-management contributed to their higher eHL regarding the COVID-19 pandemic.
**Chai [81], 2022**			
	Health information acquisition Self-management (lifestyle management)	eHL: health literacy Translational eHL: ability to apply health information	Older adults’ access to health information through social media (both directly from social media platforms and from social connections maintained by social media platforms) and self-management using this information promotes their eHL.
**Tan et al [88], 2022**			
	Health information trust	Translational eHL: decision-making and applied behaviors based on health information	Older adults who are more trusting of social media and have limited access to other sources of information are likely to make poor health decisions, suggesting that inaccurate information on social media may hinder translational eHL among older adults.
**Chen et al [85], 2021**			
	Health intervention (online group interventions)	eHL: health literacy and health self-efficacy Translational eHL: applying health literacy for problem-solving and self-management	Social media–based, online group health interventions can improve eHealth literacy among older adults, as evidenced by increased health literacy, health self-efficacy, and translational eHL.
**Wu and Yu [14], 2021**			
	Self-management (medical and lifestyle management) Health prevention Platform preference	Functional eHL and Translational eHL: ability to use online communities for health purposes Functional eHL: perceived ease of use	Social media promotes the ability of older adults to familiarize themselves with digital technology and use digital tools for health prevention and self-management by linking their online social connections; that is, it promotes their functional eHL and translational eHL. At the same time, older people prefer the perceived ease of use of digital technologies and are more inclined to adopt familiar social media platforms, which lowers their threshold for digital technology adoption, that is, increases their functional literacy.
**Ubolwan et al [84], 2020**			
	Frequency of social media use	eHL	The more frequently older adults use social media, the higher their level of eHL.
**Tennant et al [78], 2015**			
	Frequency of social media use Health information acquisition Social support (general) Health information evaluation	eHL Functional eHL: computer literacy Critical literacy: media literacy	The use of social media, the social support provided by social media, and social media–based health information behaviors can improve eHealth literacy among older adults, especially finding and evaluating health information can improve their functional literacy and critical literacy.

**Table 5:** Statistics on social media use behaviors affecting eHealth literacy among older adults (N=16).

Social media use behaviors			Studies		Studies, n (%)
**Social media use habits**					4 (25)
	Frequency of social media use	Vitolo et al [87], 2023; Ubolwan et al [84], 2020; and Tennant et al [78], 2015		3 (19)	
	Platform preference	Wu and Yu [14], 2021		1 (6)	
**Health information behaviors**					7 (44)
	Health information acquisition	Ye [80], 2024; Jang et al [82], 2023; Liu et al [44], 2022; Chai [81], 2022; and Tennant et al [78], 2015		5 (31)	
	Health information sharing	Ye [80], 2024 and Kachentawa [13], 2023		2 (12)	
	Health information evaluation	Kachentawa [13], 2023 and Tennant et al [78], 2015		2 (12)	
	Health information trust	Tan et al [88], 2022		1 (6)	
	Frequency of health information use	Ye [80], 2024		1 (6)	
**Self-management**					5 (31)
	Health prevention	Kachentawa [13], 2023; Chai [81], 2022; Liu et al [44], 2022; and Wu and Yu [14], 2021		4 (25)	
	Lifestyle management	Ye [80], 2024 and Chai [81], 2022		2 (12)	
	Medical management	Wu and Yu [14], 2021		1 (6)	
**Health interventions**					2 (12)
	Online group interventions	Chen et al [81], 2021 and Lee and Ryu [83], 2023		2 (12)	
**Telemedicine**					2 (12)
	Communicating with health care providers	Oh et al [86], 2023		1 (6)	
	Accessing health care services	Liu et al [44], 2022		1 (6)	
**Health decision-making**					1 (6)
	Medical decision	Kachentawa [13], 2023		1 (6)	

##### Social Media Use Habits

Social media use habits are themes extracted based on older adults’ general social media use behaviors, which mainly concern older adults’ social media use frequency and platform preferences. Of the 16 included literature, 4 (25%) papers discussed the social media use habits of older adults, 3 (19%) focused on the frequency of social media use by older adults, and 1 (6%) focused on older adults’ preferences for social media platforms. The findings indicated a substantially positive correlation between social media use patterns and eHealth literacy.

The most prominent manifestation was that increased use of social media increased the eHealth literacy of older individuals. Ubolwan et al [84] included social media use in assessing the level of eHealth literacy and its correlates in the older population in Thailand. This research revealed a statistically significant positive correlation between the duration of internet or online social media use among older people and their eHealth literacy status. Similarly, Tennant et al [78] discovered that, compared to those who seldom use social media, individuals who used social media regularly had better eHealth literacy. Vitolo et al [87] discussed the adverse effects of reduced frequency of social media use on eHealth literacy in older adults from the opposite perspective, that is, frail older patients had limited use of digital technology and the internet and poorer digital health literacy.

In addition, the study by Wu and Yu [14] was the only study that specifically examined older adults’ preferences for social media platforms. Their findings revealed that the most commonly used social media feature among older adults was instant text messaging. Unlike younger generations, who tended to use a wide variety of apps, older adults preferred a limited selection of platforms, often opting for those they were already familiar with. The study also highlighted that older adults place greater emphasis on the perceived ease of use of eHealth technologies rather than their practicality.

##### Social Media Health Information Behaviors

Health information behavior on social media was the most frequently discussed social media activity influencing older adults’ eHealth literacy. This behavior encompasses various aspects, including health information–seeking, sharing, evaluation, trust, and use frequency. Studies consistently suggest that older adults’ health information behaviors on social media positively impact their eHealth literacy, particularly functional eHealth literacy, critical eHealth literacy, and translational eHealth literacy.

In particular, Ye [80] thoroughly assessed the influence of older individuals’ behaviors of using health information on social media. The study examined the correlation between 4 aspects of older persons’ online health information use habits, including social media (frequency of using online health information, forwarding and sharing online health information, using online health information to regulate one’s behavior, and positive attitudes toward online health information), and 3 dimensions of eHealth literacy (the ability to apply eHealth literacy, the ability to make critical judgments and decision-making ability) [80]. A notable positive association was observed among the 7 variables, namely, stronger habits of internet use of health information among older persons corresponding to better eHealth literacy. Among these practices, the dissemination and interchange of internet health information among older people had the most significant influence on their eHealth literacy. Moreover, the habits of older individuals’ internet health information use had a significant impact on older people’s health decision-making ability. The study further affirmed that older individuals’ adoption of social media to browse health content was an effective path to enhance their eHealth literacy because they would improve their understanding of health information in various ways during browsing to regulate their health behaviors.

Kachentawa [13] further recognized the important positive impacts of social media use on older individuals’ eHealth literacy and suggested that 2 important literacies related to social media should be included in the system of indicators of older people’s eHealth literacy in Thailand within the setting of the new normal lifestyle. These 2 indicators include media and information literacy and computer and social media literacy. The former elucidates the capacity of older Thai individuals to assess the reliability of the COVID-19 pandemic information shared on social media platforms and to comprehend tactics and demonstrations of technology associated with social media. The latter focuses on the capacity of older individuals to acquire new knowledge on social media, including the ability to use relevant apps, communicate with others, obtain accurate information regarding the COVID-19 pandemic, and resolve fundamental challenges encountered on social media. It can be concluded that these 2 indicators encompassed older adults’ behaviors related to understanding, evaluating, sharing, and seeking health information on social media. This further underscores the significant impact of health information behaviors on social media in shaping eHealth literacy among older adults.

The increased social media–based health information behaviors among older adults were also found to help them improve their eHealth literacy. Tennant et al [78] analyzed social media health information–seeking behaviors and noted that using social media to obtain and evaluate eHealth information contributed to their eHealth literacy, especially for functional eHealth literacy and critical eHealth literacy. The study conducted by Liu et al [44] investigated older individuals’ eHealth literacy during the COVID-19 epidemic. The findings demonstrated that more than half of the older adults had better eHealth literacy during this period. The study also identified several key factors, such as advancements in IT and efforts in epidemic prevention and control, that enabled older adults to effectively use digital technologies, including social media.

In addition, social media impacts the eHealth literacy of older adults by expanding their sources of health information. The study by Chai [81] indirectly demonstrated the impact of social media on improving older individuals’ eHealth literacy by exposing how social media facilitated their access to health information. This study pointed out that social media enhanced health literacy among older adults by providing them with direct health information, on the one hand, and helping them to access health content and health promotion resources from social networks by maintaining social connections, on the other hand. Jang et al [82] supported this view from the opposite perspective. Their study found that older adults who had less access to digital resources, among others, had restricted health sources and exhibited lower preventive behaviors that were needed to enhance their eHealth literacy.

Finally, it is worth noting that older adults’ trust in the sources of health information on social media can significantly influence their translational eHealth literacy. Tan et al [88] highlighted that older adults who exhibited greater trust in social media and had limited access to alternative information sources were more likely to make suboptimal health decisions. This finding suggests that the presence of inaccurate information on social media may impede the development of translational eHealth literacy among older adults.

##### Self-Management

Older adults’ self-health management behaviors on social media, including health prevention, medical management, and lifestyle management, had a positive impact on their eHealth literacy, particularly their translational eHealth literacy. Given that some (12/16, 75%) studies were conducted in the context of the COVID-19 pandemic, they specifically highlighted how older adults’ use of health information for health prevention influenced their eHealth literacy [13,44,81].

Kachentawa [13] proposed a media and information literacy indicator within the framework of eHealth literacy for older adults in the “new normal.” This indicator highlighted the ability of older adults in Thailand to effectively use health information from social media to protect themselves from COVID-19 infection. Similarly, focusing on older adults during the COVID-19 pandemic, Wu and Yu [14] explored the use of social media as a grid-based management platform during this period. This platform reconnected older adults to an online model of a familiar social network, enabling them to become accustomed to digital technologies and access eHealth services. It not only supported health prevention efforts but also facilitated the management of their medical needs.

Liu et al [44] noted that the normalization of the pandemic prevention and control measures encouraged older adults to adopt electronic devices and ITs for health management and prevention, thereby enhancing their eHealth literacy. Chai [81] focused on how older adults applied health information obtained from social media to their daily practices during the COVID-19 pandemic, improving their health behaviors and outcomes. This process, in essence, represents an enhancement in their translational eHealth literacy.

Furthermore, Ye [80] explored the mechanisms through which older adults enhanced their eHealth literacy via self-management on social media. The study highlighted that, through the process of acquiring health information on social media platforms, older adults gained increased health knowledge, improved their health awareness, and developed stronger problem-solving abilities related to health issues. These advancements enabled them to effectively use online health knowledge for self-management, such as regulating their own health behaviors. In addition, this process fostered a heightened sense of health self-efficacy, ultimately leading to improved eHealth literacy.

##### Health Interventions

In total, 13% (2/16) of health intervention studies conducted on social platforms had validated the efficacy of social media in augmenting eHealth literacy among older individuals [83,85]. Lee and Ryu [83] incorporated social media into a walking program for older adults, which included mobile medical devices and gamification elements. Findings showed that participants’ eHealth literacy improved, and the positive impact of social media was generally appreciated by older adults. Similarly, the study by Chen et al [85], while not directly measuring changes in older adults’ eHealth literacy levels after intervening in older adults’ health through social media, quantified substantial improvements in health literacy and overall health self-efficacy while also noting a rise in older persons’ understanding of the significance of health, as well as their capacity to address health issues.

##### Telemedicine

Social media platforms hold significant potential as telemedicine platforms, facilitating communication between older adults and health care providers while helping them access online health services. Only 13% (2/16) of studies had discussed the role of social media as a telemedicine platform in enhancing eHealth literacy among older adults [44,86]. Oh et al [86] revealed that Asian Americans improved their confidence and capabilities by communicating with health care providers through online platforms, such as social media, thereby enhancing their health information literacy. This finding reflects the potential of social media communication with health care providers to promote communication literacy among older adults. Furthermore, during the COVID-19 pandemic, the role of social media in telemedicine was effectively demonstrated. Liu et al [44] found that home isolation measures during the COVID-19 pandemic, which restricted in-person medical consultations, encouraged older adults to adopt telemedicine practices. These online health behaviors played a critical role in improving their eHealth literacy during the COVID-19 pandemic.

##### Health Decision-Making

Health decision-making emphasizes the specific decision-making behaviors of older adults based on health information obtained from social media, fundamentally reflecting the application of health information. This corresponds to the dimension of translational eHealth literacy. Kachentawa [13] identified media and information literacy as a key indicator of older adults’ eHealth literacy in the new normal. This indicator included their health decision-making abilities, particularly their capacity to use health information from social media to make treatment decisions after contracting COVID-19. This, to some extent, reflects the translational dimension of eHealth literacy.

#### Impact of eHealth Literacy on Older Individuals’ Social Media Health Behaviors

According to the results, eHealth literacy, in turn, affects older adults’ performance of health behaviors on social media. Of the 16 studies, 8 (50%) were involved, and the details are provided in Table 6. The most discussed dimensions of eHealth literacy were critical eHealth literacy (4/16, 25%) and functional literacy (4/16, 25%).

**Table 6:** The impact of eHealth literacy (eHL) on older adults’ social media behaviors.

Study	Theoretical mapping: dimensions of eHL	Influenced social media health behaviors	Mechanism of association and key conclusions
Ye [80], 2024	eHL	Health information acquisition	The level of eHealth literacy among older adults is directly proportional to the diversity of their access to information.
Matchanova et al [51], 2023	Functional eHL and critical eHL: verbal IQ, numeracy skills, knowledge	Health information sharing Health information evaluation	Older adults who lack functional literacy and critical eHL inaccurately assess health information on social media and are more likely to share incorrect health information.
Wang and Zhang [42], 2023	eHL: digital literacy Communicative eHL: interactive health literacy Critical health literacy	Health information acquisition	eHL was positively associated with older adults’ social media health information acquisition behaviors, as evidenced by communicative eHL predicting older adults’ passive and long-term health information gathering behaviors and critical eHL predicting older adults’ active information acquisition behaviors.
Liu et al [44], 2022	Functional eHL: barriers to the use of digital technology Critical eHL: distinguish health information	Health information acquisition Telemedicine (accessing health care services) Health information evaluation Health decision-making	Older adults who lack functional eHL have difficulty in accessing health care services through social media, and the lack of critical eHL makes it difficult for them to properly evaluate health information and make sound health decisions.
Chai [81], 2022	Critical eHL: understanding and evaluation of health information Functional eHL: know little about new digital technologies	Health information evaluation Frequency of social media use	Older adults who lack critical literacy struggle to understand the scientific logic of health rumors on social media and tend to reject new communication technologies, such as social media, due to a lack of digital literacy.
Tan et al [88], 2022	Critical eHL: trust in health information sources	Health decision-making (medical decisions)	Lack of critical eHL diminishes the ability of older adults to make sound health decisions.
Wu and Yu [14], 2021	Functional eHL: unfamiliarity with the new digital technology	Telemedicine (accessing health care services)	Lack of functional eHL affects attitudes and willingness to adopt telemedicine among older adults.
Shang and Zuo [79], 2020	eHL	Health knowledge learning	Self-efficacy and perceived benefits partially moderate the relationship between eHealth literacy and willingness to learn about health.

First, critical eHealth literacy directly affects older adults’ assessment of social media health messages. Matchanova et al [51] discovered that persons with little knowledge and fundamental numeracy abilities might have had impaired accuracy in assessing the truthfulness of news headlines about the COVID-19 pandemic on social media and were more prone to disseminating false information on social networks. Chai [81] found inconsistencies in older adults’ understanding and evaluation of the COVID-19 pandemic–related rumors. While some older adults were able to identify misinformation about the pandemic, they often lacked an understanding of the underlying scientific logic. This highlights a gap in critical eHealth literacy among older adults.

Second, a lack of critical eHealth literacy can also affect older adults’ ability to make sound health decisions. Tan et al [88] noted that older adults’ trust in social media disinformation affected their hesitancy to take a new coronary vaccine. Although the findings showed that social media only slightly predicted vaccination intention among older adults, the study also found that trusting only formal sources and distrusting informal sources was associated with higher vaccination intentions. Therefore, improving older individuals’ ability to discern health information on social media can eliminate their trust in false health information, thereby shaping their health behaviors.

Similarly, Liu et al [44] observed that older persons predominantly relied on information to avoid COVID-19 infection and to enhance their well-being. They also highlighted that their limited capacity to understand and assess health-related information hindered their ability to distinguish between reliable information on social media, thus impacting their capacity to make informed health choices [44]. Similarly, critical eHealth literacy is essential for older people to effectively access health information on social media. Insufficient skills can hinder accurate information assessment, increase vulnerability to misinformation, and complicate the health decision-making process.

Furthermore, research has revealed a correlation between functional literacy and the inclination of older populations to use social media platforms (3/16, 19%) [14,80,81]. Ye [80] demonstrated that older people who relied heavily on online health information exhibited a more favorable and hopeful outlook toward their health. Moreover, they were more inclined to use internet-based platforms, such as social media, to obtain health-related information. In contrast, Chai [81] argued from the opposite perspective that older adults who lacked functional eHealth literacy showed rejection of new communication technologies, such as social media, as most of them have had unpleasant experiences with the adoption of new technologies. Similarly, Wu and Yu [14] found that the lack of functional eHealth literacy affected attitudes and willingness to adopt telemedicine through social media among older adults.

Finally, eHealth literacy directly affects older adults’ health information behaviors [42,51,79]. Matchanova et al [51] revealed that eHealth literacy also affected health information sharing behaviors. The study conducted by Shang and Zuo [79] primarily aimed to investigate the inclination of older individuals to acquire knowledge about health through the use of social media platforms. Using self-efficacy and perceived benefits as mediating variables, they examined the effect of eHealth literacy as a cognitive state on older people’s willingness to acquire health knowledge through social media. Research indicated that older individuals who possessed greater eHealth literacy tended to view themselves as having greater self-efficacy and perceived benefits in their health-related knowledge. Self-efficacy and perceived benefits exerted a minor moderating effect on the association between eHealth literacy and the inclination of older individuals to acquire knowledge about health via social media.

Wang and Zhang [42] found that older adults usually browsed health information passively, while those with higher digital literacy were more likely to actively seek health information online. In addition, positive correlations were observed between interactive literacy and functional literacy in health literacy and the health information acquisition habits of older persons. Thus, higher eHealth literacy among older people led to more proactivity and improved performance in using social media to access eHealth information.

### Factors Influencing Older Adults’ Use of Social Media to Improve eHealth Literacy

#### Overview

**Figure 4:**
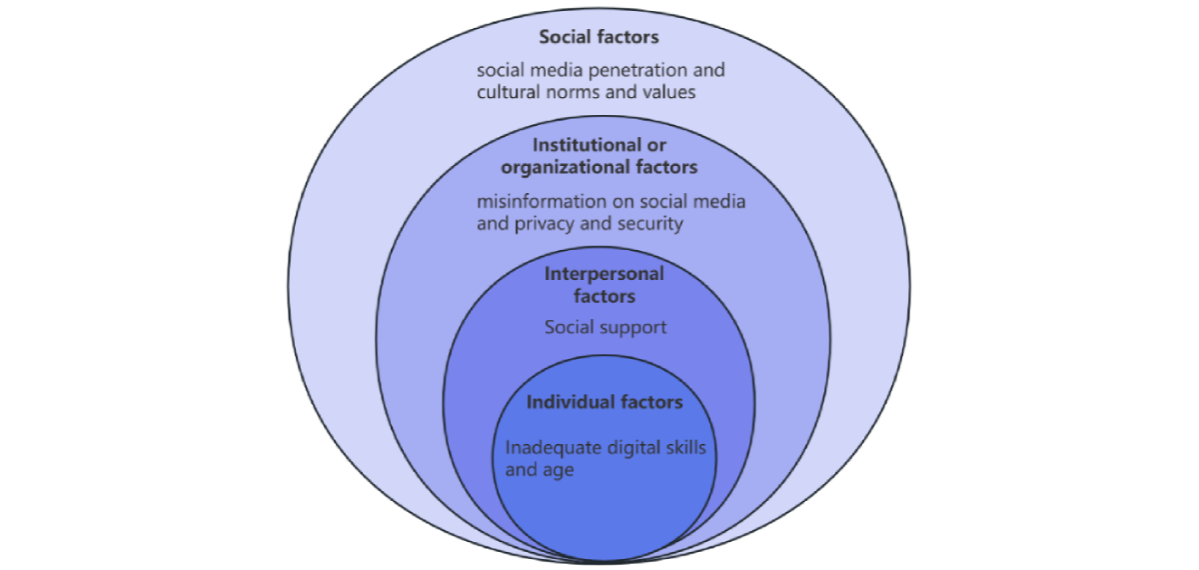
Framework diagram of the influencing factors on older persons’ use of social media to enhance their eHealth literacy. This figure categorizes key factors affecting eHealth literacy, with reference sources as follows: (1) social factors: social media penetration [13,14,42,44,79,82,84,86] and cultural norms and values [14], (2) institutional and organizational factors: misinformation on social media: [13,42,44,51,78,82,88] and privacy and security [14], (3) interpersonal factors: social support [14,78,80,81,83,85], and (4) individual factors: inadequate digital skills [13,14,42,44,51,79,87] and age [14,51,87].

Previous articles dealing with the factors affecting eHealth literacy in older adults have tended to list points based on empirical results and lacked theory-guided categorization. The study by Shi et al [45] adopted a SEM to classify the factors affecting eHealth literacy among older adults, which provides inspiration for this review. In this review, the elements influencing the use of social media by an older adult to enhance eHealth literacy were classified into four main areas: (1) individual factors, including older adults’ inadequate digital skills (7/16, 44%) [13,14,42,44,51,79,87] and age (3/16, 19%) [14,51,87]; (2) interpersonal factors, involving social support (6/16, 38%) [14,78,80,81,83,85]; (3) institutional or organizational factors, including misinformation on social media (7/16, 44%) [13,42,44,51,78,82,88] and privacy and security (1/16, 6%) [14]; and (4) social factors, including social media penetration (8/16, 50%) [13,14,42,44,79,82,84,86] and cultural norms and values (1/16, 6%) [14]. Figure 4 [13,14,42,44,51,78-88] presents the framework of these factors.

#### Individual Factors

In the context of SEM, the individual level refers to individual characteristics [89,90], including knowledge and skills, attitudes and beliefs, demographic factors, and self-efficacy [90-92]. Inadequate digital skills among older adults and age are identified as individual factors in this review**.**

##### Inadequate Digital Skills Among Older Adults

The prevalence of inadequate knowledge and proficiency in using digital devices among older people poses a significant obstacle to their eHealth literacy. The first obstacle is the reluctance of older individuals to use social media to acquire health information and services. Ubolwan et al [84] contended that the limited social media abilities of older persons might result in hesitancy to use social media for eHealth participation. This, in turn, could negatively influence older people’s eHealth literacy, as there exists a direct relationship between the frequency of social media use and eHealth literacy. Wu and Yu [14] pointed out that older people are deprived of lifelong learning environments and have relatively low confidence in digital technology after retirement. A prominent manifestation is that, unlike the younger generation, who care about the practicality of digital health care, older people are more concerned with the perceived ease of use of digital technology [14]. Moreover, the lack of digital experience leaves older adults with high health management needs facing low technology adoption. Second, it hinders older adults’ effective access to health resources. Chai [81] noted that older adults’ lack of skills in using digital technology can make them face challenges in health information retrieval. Similarly, Wang and Zhang [42] argued that older individuals with little digital literacy are less able to access health information via the internet or technological devices.

Furthermore, the lack of proficiency in digital skills among older individuals can result in the digital divide among them extending to eHealth, therefore impeding the achievement of health equity [13,42,78]. Kachentawa [13] argued that perceived self-efficacy and digital competence affected older adults’ adoption of digital technology. While perceived self-efficacy is primarily related to older people’s enjoyment of using digital technology, the core problem remains in older adults’ lack of digital skills. This issue is reflected in the indicators developed by the author related to the eHealth literacy of older persons, namely computer and social media literacy [13]. It suggests that in the new normal, older adults must not only have the knowledge and skills to use digital devices, such as computers and social media, but also have the ability to solve basic problems with social media. Tennant et al [78] highly emphasized the important role of digital literacy (computer literacy) in the older adult population, arguing that lower digital literacy among older adults prevents them from truly benefiting from health care innovations and may reopen the digital divide, exacerbating current health disparities.

##### Age

Another individual factor is age. However, the results of the studies were not consistent. While the study by Wu and Yu [14] identified aging as a significant determinant of older people’s digital technology use and their attitudes toward telemedicine, Vitolo et al [87] found that digital literacy was only partially influenced by age. The role of age was further weakened in the study by Matchanova et al [51], where the findings did not demonstrate a substantial impact of age on the probability of sharing false or accurate information online. Nevertheless, it was observed that this phenomenon could be attributed to the age of the study participants (aged 50 years), but most of the significant effects on susceptibility to misinformation and sharing were centered at the age of 65 years. Thus, there may be differences in perceived susceptibility to social media misinformation about health by age.

#### Interpersonal Factors

The interpersonal dimension of SEM emphasizes the influence of social networks and social support systems on individual behaviors [89-91]. In this review, social support plays a crucial role in enabling older people to use social media to enhance their eHealth literacy.

The social supportive role of social media is manifested first and foremost in increasing the health information resources available to older adults by linking their relationships on online platforms. Social relationships, the traditional way for older individuals to obtain health information or resources, have achieved rapid dissemination on online platforms with social media connections. This model played an important role during the COVID-19 pandemic. Wu and Yu [14] noted that during the COVID-19 pandemic, social media platforms, such as WeChat, expanded offline community management to online, creating a new model of online acquaintance relationships and, therefore, continued maintaining health support through social networks. Chai [81] also noted that older adults accessed health information through online social relationships during the COVID-19 pandemic.

Furthermore, the effect of social support is reflected in the interactive advantages of social media in facilitating health communication and health information sharing behaviors among older adults. Tennant et al [78] argued that the greater social support that older adults derive from social media is based on stronger Web 2.0-connected support networks and the multifunctionality factor of social media. Older adults can enhance their understanding of their health status and connect with online friends with similar experiences through social support venues built on interactive social media. Ye [80] further clarified that older adults’ online health information sharing and exchange behaviors had the greatest impact on their eHealth literacy, outweighing the effect of the other dimensions (frequency of use of online health information, use of online health information to regulate their behaviors, and the impact of favorable attitudes toward online health information) on eHealth literacy.

Finally, the facilitating role of social support provided by social media is reflected in the strength of social media as a medium for group-based health interventions. Of the 16 studies, 2 (13%) social media–based health intervention studies found that social media was effective in motivating older adults to participate in health intervention programs [83,85]. Lee and Ryu [83] found that mHealth apps incorporating social media allowed older adults who were socially isolated during the COVID-19 pandemic to feel socially connected again, as they shared and interacted with their peers and encouraged them to use these apps. The rationale for the finding was explained in more detail in the study by Chen et al [85]. They pointed out that interacting with others is a key incentive for older adults to engage in health interventions. Older adults enjoy sharing experiences and opinions in online discussions on social media and, over time, they develop a sense of socialization that enhances feelings of connectedness and engagement.

#### Institutional or Organizational Factors

##### Overview

On the basis of the SEM, institutional or organizational factors refer to the influence of institutional or organizational settings individuals are embedded in on personal behaviors [89,90]. In the health context, organizational structures and processes, as well as the environment within the organization, including safety, have an impact on people’s health behaviors [90]. This review revealed that misinformation on social media and privacy and security concerns associated with social media pose possible obstacles that hinder older people from enhancing their eHealth literacy using social media.

##### Misinformation on Social Media

The issue of information credibility on social media has attracted a great deal of attention from researchers, who have argued that the proliferation of false information on social media is a serious threat to effective access to health information by older adults [13,42,78,79]. This was especially true during the COVID-19 pandemic, when false news on social media was rampant and caused great harm to older adults [13] and affected their health decision-making abilities and health behavioral norms. For example, studies by Jang et al [82] and Tan et al [88] found that trust in misinformation on social media increased new coronary vaccine hesitancy in older adults. Social media also contributes to the dissemination of misinformation about health among older adults. Matchanova et al [51] noted that the social media sharing function does not require the involvement of subjective agency other than motivation, which may influence the reflective accuracy of older adults to spread misinformation about health.

Part of the literature identifies the lack of older adults’ ability to assess social media information, such as media literacy or information literacy, that is, the ability to assess the trustworthiness of social media. Kachentawa [13] included this competency as one of the indicators of eHealth literacy among the new normal older adults and emphasized the importance of assessing the trustworthiness of the information in the context of social media to improve eHealth literacy among older adults. Similarly, Tennant et al [78] noted a real lack of confidence among older adults in assessing the quality of social media health information. Furthermore, Liu et al [44] found that older adults had the lowest information judgment scores. Nonetheless, Wang and Zhang [42] revisited the relationship between key health literacies related to the ability to assess health information and older adults’ health information acquisition behaviors from the perspective of the motivation of older adults’ health information acquisition behaviors. The study yielded a different and interesting finding that critical health literacy was not significantly correlated with health information acquisition behavior; that is, even if older adults are unable to critically analyze health information, they are able to facilitate their health information access behaviors as long as they have basic literacy and communication skills.

##### Privacy and Security

Wu and Yu [14] identified financial security as a barrier that hinders older persons from the adoption of digital services. Older adults are anxious about the potential loss of money through the use of online payments.

#### Social or Community Factors

##### Overview

The social or community level in SEM refers to societal influence and cultural or subcultural norms and values [89]. Regarding social influence, the development of social media technology can affect personal health behaviors. In this review, this influential factor is identified as social media penetration. Meanwhile, the influence of cultural norms was reflected in a study in China [14].

##### Social Media Penetration

The high penetration of social media among older adults is a notable trend that has been the focus of most studies, and this trend’s development establishes a crucial basis for the function of social media in enhancing eHealth literacy among the older population. Given the growing population of older individuals using social media, it has become a crucial medium for them to obtain and exchange health-related information [13,44,82] and creates novel avenues for older individuals to acquire health advice [79]. This high penetration is mainly achieved through 2 aspects: one is driven by IT (5/16, 31%) [42,44,79,84,85], and the other is catalyzed by the COVID-19 pandemic (2/16, 13%) [13,44]. One consequence of the swift advancement of IT is the establishment of a technological framework that enables older people to use social media. Various studies have indicated an increase in the frequency of social media use among older adults in recent years [44,79,84,85]. Moreover, this IT push is inadvertently creating social pressure on older people to learn to use digital technology tools [14]. Furthermore, the outbreak and normalized management of the COVID-19 pandemic further increased the adoption of social media by older adults. During the COVID-19 pandemic, older adults faced physical health risks and social isolation situations, and social media became not only a tool for them to maintain social relationships but also an important platform for them to obtain health-related information and health services [13,44].

The high penetration of social media is also evident in the significant expansion of channels for accessing health information, which has transformed the ways they obtain it. Ye [80] pointed out that social media can bring more health channels to older adults and increase their access to health information. Furthermore, Oh et al [86] argued that social media can serve as an effective channel to supplement older patients’ health information and knowledge when they have poor communication with their health providers, thus improving their communicative eHealth literacy.

##### Cultural Norms and Values

Wu and Yu [14], in the study of older adults in Wuhan, China, found that China’s social culture of acquaintance profoundly influenced older adults’ adoption of digital health care. Chinese older adults were more likely to adopt programs based on acquaintance relationships and traditional health care processes than those in other countries. The acquaintance relationship in Chinese society is a distinct cultural phenomenon characterized by the dichotomous connection between acquaintances and the line between insiders and strangers [14]. During the COVID-19 pandemic, the “grid management” model facilitated through social media chat groups linked a new type of online acquaintance relationship that could help manage the health care needs of older adults by providing them with a sense of trust and setting common rules. To this end, their study indicates the need to take into account the cultural environment while encouraging the acceptance of digital health among older individuals.

## Discussion

### Principal Findings

This study is the initial systematic literature review that investigates the correlation between social media use behaviors and eHealth literacy in the older adult population. The increasing global aging population, the proliferation of digital technologies, and the COVID-19 pandemic have significantly immersed older adults in the social media and eHealth phenomenon. This situation offers them both prospects and obstacles to fully benefit from digital health technologies. On the one hand, the advantages of social media have expanded the channels for older adults to engage in eHealth and, on the other hand, older adults’ eHealth literacy greatly influences their willingness to adopt social media, as well as their performance of health behaviors based on social media platforms.

To further integrate older adults into the eHealth trend and achieve active aging, it is essential to establish a positive feedback loop between social media behaviors and eHealth literacy. This involves enabling older adults to gradually enhance their eHealth literacy through health-related practices on social media, which, in turn, improves their social media health behaviors and experiences, ultimately bridging the digital divide. Therefore, this study aimed to synthesize and analyze recent research on this relationship, uncovering patterns and influencing factors to identify both the potential and barriers of using social media to enhance eHealth literacy among older adults. The findings are expected to inform targeted eHealth literacy interventions for this population, fostering a sustainable cycle of mutual reinforcement between health practices and health skills.

We extracted themes related to older adults’ social media behaviors and the dimensions of their eHealth literacy for cross-analysis. The findings reveal that older adults’ social media behaviors and eHealth literacy are mutually influential and interactive. Specifically, this review provides comprehensive answers to the RQs.

Regarding the first RQ, we identified that older adults’ social media behaviors encompass general use behaviors and health-related behaviors. General use behaviors include habits such as use frequency and platform preferences, while health-related behaviors involve a range of activities aimed at health purposes, such as health information behaviors, health decision-making, self-management, participation in health interventions, and engagement in telemedicine. Among these, health information behaviors are the most common and widely discussed health activities.

For the second RQ, most (14/16, 88%) of the reviewed studies confirmed the positive impact of older adults’ social media behaviors on enhancing their eHealth literacy. First, regarding general use behaviors, the frequency of social media use significantly improves older adults’ eHealth literacy, aligning with previous research that observed a positive correlation between the frequency of internet technology use and eHealth literacy [93,94]. In addition, familiar social media platforms enhance older adults’ perceived ease of use of digital technologies, thereby increasing their willingness to engage in health-related activities on social media [14].

Second, health-related behaviors on social media are the most effective and direct practices for improving older adults’ eHealth literacy. These practices include health information behaviors, health management, health interventions, telemedicine, and health decision-making, with health information behaviors being the most frequently (7/16, 44%) discussed. Older adults’ self-management and health decision-making activities are often built upon their understanding and application of health information obtained via social media. Furthermore, social media serves as a highly effective platform for group-based, online health interventions, where its interactive features enhance older adults’ participation in interventions and their willingness to adopt digital technologies [83,85]. The platform’s potential as a telemedicine tool, especially during the COVID-19 pandemic, has been highlighted as older adults began using social media for telemedicine and, through these practices, improved their eHealth literacy.

For the third RQ, the findings revealed that older adults’ eHealth literacy reciprocally influences their social media behaviors. This includes their willingness to adopt social media and other digital technologies [14,44,81], their ability to understand and evaluate health information on social media [44,51,88], their willingness to learn health knowledge through social media [79], and their proactivity in acquiring health information [42]. Among these, functional and critical eHealth literacy have the most significant impacts. A lack of functional eHealth literacy not only hinders older adults’ effective use of social media and other digital technologies but also reduces their willingness to adopt them. A deficiency in critical eHealth literacy affects their ability to understand and evaluate information, which further impacts their health decision-making and self-management behaviors.

By integrating the insights from RQs 2 and 3, we conclude that older adults’ eHealth literacy and their social media behaviors exhibit a reciprocal relationship, highlighting the need for interventions that leverage this dynamic to enhance both dimensions.

For the fourth RQ, older adults’ use of social media for health participation is influenced by individual, interpersonal, institutional or organizational, and social factors. At the individual level, inadequate digital skills [13,14,42,44,51,79,87] and age-related challenges [14,51,87] limit their ability to access and use social media effectively, contributing to the digital divide. Interpersonal factors, such as social support [14,78,80,81,83,85], enhanced health information acquisition or sharing and participation in health interventions, which are vital for improving eHealth literacy. Institutional barriers, including misinformation [13,42,44,51,78,82,88] and privacy concerns [14], further hinder their engagement, emphasizing the need for critical eHealth literacy. Moreover, the high penetration of social media, driven by technological advancements and the COVID-19 pandemic, has expanded access to health information and services, offering new opportunities to bridge these gaps [13,14,42,44,79,82,84,86]. Finally, promoting the use of eHealth technologies among older adults also requires consideration of cultural contextual factors at the societal level [14].

### Considerations for Establishing a Reciprocal Mechanism Between Social Media Health Practices and Older Adults’ eHealth Literacy

#### Overview

The findings reveal the immense potential of social media to enhance older adults’ eHealth literacy. To further promote their active use of social media for health interactions, information acquisition, and service use, it is essential to enable older adults to continually improve their eHealth literacy through social media–based health practices. This improvement, in turn, can lead to positive feedback in their social media use experiences, ultimately establishing a reciprocal reinforcement system between social media use and eHealth literacy. To this end, theoretical and practical efforts can be made in the following areas.

#### Leveraging Postpandemic Momentum to Enhance eHealth Literacy Among Older Adults Through Social Media

The high penetration of social media and the catalyst of the COVID-19 pandemic have facilitated changes in older adults’ technology’s use behaviors and health behaviors, laying the groundwork for improving older adults’ eHealth literacy. The continuous development and popularization of social media have revolutionized the traditional pattern of health information dissemination [95,96] and changed the habits of older adults in accessing eHealth information or services [44,97]. Especially with the COVID-19 outbreak, this pattern has deepened further. On the one hand, the social blocking measures forced older adults to rely on social media for information and lifestyle services, as well as social support, among others [98]. On the other hand, the epidemic posed multiple risks to older adults [99-101], who were forced to step out of their comfort zones and start using social media and engaging in eHealth activity practices. The COVID-19 pandemic is arguably a major reason for the rise in social media use among older adults [13]. Moreover, during this period, older adults showed unprecedented enthusiasm for learning about eHealth tools [102]. Interest is an important reason for improving eHealth literacy [103]. Therefore, the present moment, that is, after the end of the COVID-19 pandemic and in the context of older people’s heightened experience of and interest in eHealth technologies, as well as social media, is an optimal time to improve eHealth literacy among older people. Countries around the world should take this opportunity to lay and plan to encourage older adults to use social media more to participate in eHealth activities and to accelerate the pace of active integration of older adults into the trend of eHealth technology.

#### Reconsidering the Dimensions of eHealth Literacy Among Older Adults in the Context of Web 2.0

Findings regarding the impact of eHealth literacy on older adults’ social media health behaviors bring more serious practical considerations to the achievement of this goal. According to the review, eHealth literacy among older adults can, in turn, affect older adults’ social media use behaviors and performance, including influencing older adults’ understanding and evaluation of social media health information [44,51,88], willingness to use social media to access or learn health information [79-81], and access to health information initiative [42]. More importantly, the lack of digital skills and misinformation on social media, the 2 prominent factors affecting older adults’ use of social media to improve eHealth literacy, are closely related to 2 important components of eHealth literacy: digital literacy and critical health literacy, respectively. The lack of these 2 literacies not only prevents older adults from truly benefiting from eHealth but also has the potential to exacerbate health disparities by extending the digital divide into the eHealth domain [42,79]. Kachentawa [13] innovatively included social media literacy in the indicators of eHealth literacy for older adults in the new normal. In his opinion, social media literacy among older adults included not only the ability to assess information but also the skills in understanding, using, and dealing with problems in social media. However, there is still no consensus on the concept of social media literacy. Some scholars define the concept of social media literacy as encompassing the technical and practical skills required for using social media [104,105], while others prefer to use the concept of media literacy as a substitute for social media literacy [106,107]. As the use of social media among older adults increases, social media literacy should be further emphasized as a component of eHealth literacy among older adults. In addition, relevant interventions should be centered on how to adapt functional and critical eHealth literacy for older adults.

#### Actively Developing Age-Friendly Integrated Social Media Health Service Platforms

Self-efficacy has been confirmed by previous research to be positively associated with older adults’ willingness to use digital health technology [108]. In turn, self-efficacy in older adults was positively associated with the perceived ease of use of eHealth technology [109]. Thus, digital literacy among older adults can be achieved by lowering the threshold of eHealth technologies. In addition to further strengthening IT infrastructure, efforts should be made to actively develop social media health service features and digital tools tailored to the needs of older adults.

Existing research on intelligent health and medical services for older adults has extensively investigated their needs, with a primary focus on developing diverse mHealth apps. For instance, Zhou et al [110] developed a home health management app tailored to the needs of older users, which promoted self-health management among this population. Similarly, Blinka et al [111] designed a sensor-based mobile app to help older adults assess frailty. However, this review reveals that older adults’ digital platform preferences significantly influence their willingness to adopt technology. Wu and Yu [14] highlighted that older adults prefer limited and familiar digital platforms, which enhance their perceived ease of use and, consequently, their willingness to engage in health activities via social media. In addition, among the 16 included studies, 2 (13%) articles focusing on health interventions that used specialized health platforms. Although the intervention results showed improvements in older adults’ eHealth literacy, there remains a potential risk of low adoption willingness if these interventions are to be widely implemented among the older population.

Older adults who have experienced the COVID-19 pandemic are already familiar with social media platforms, and further investigation into their preferences for these platforms is warranted. Aging impacts cognitive abilities, leading to slower reactions and increased cognitive vulnerability [112,113]. The proliferation of mobile apps can negatively affect their perceived ease of use. Therefore, effectively leveraging the social media platforms that older adults are already familiar with to create an integrated mHealth platform—incorporating features such as health information dissemination, online consultations, health monitoring, and interactive support—can enhance their willingness to adopt and use these technologies. For example, Samad-Soltani et al [114] proposed integrating Telegram-based smart bots into health care, which not only minimizes the need for app installations but also supports a variety of health-related tasks while fostering interaction with contacts. Another example is the hospital WeChat official account in China, which is a platform for embedding the hospital’s eHealth system into WeChat, China’s most popular social media, to facilitate older adults’ access to health care information and services [79,115]. In the future, the integration of mHealth and social media is expected to become a new generation of eHealth trends, and related studies can pay more attention to this trend.

Last but not least, the development of a social media health integration platform should take into account other factors that influence the adoption of technology by older adults. Although the influences of privacy and security, age, and culture were not widely discussed in the included literature, they still play an important role in previous work on factors affecting eHealth literacy among older adults [45,116]. Therefore, future research should focus on the influence of these factors on older adults’ use of social media for eHealth engagement. Previous studies have found that issues of privacy and security have been widely identified as a barrier to older adults’ use of digital health technologies [109,117], and social media amplified this challenge [38]. Accordingly, the resolution of privacy and security issues will potentially have a direct impact on older adults’ willingness to use social media to engage in eHealth. In addition, age, as well as cultural factors, may affect older adults’ attitudes toward using social media to enhance eHealth literacy. Relevant technology development should also fully consider the characteristics and needs of older adults at different age stages and in different cultural contexts.

#### Optimizing Social Media for Engaging and Reliable Health Information for Older Adults

The findings indicate that health information behaviors on social media are the most common and direct form of health practice for enhancing eHealth literacy among older adults. Behaviors, such as self-management and health decision-making, are often built upon their understanding and evaluation of the health information or knowledge they acquire. Previous studies have highlighted that the type and source of information are crucial contextual factors influencing older adults’ online health information–seeking behaviors [118,119]. However, in this review, as most studies were published during the COVID-19 pandemic, there is a predominant focus on older adults’ acquisition and application of COVID-19 pandemic–related information, with limited attention to other types of health information. Future research should place greater emphasis on older adults’ preferences for other health information types. In addition, the review reveals that trust in health information sources significantly influences older adults’ health decisions [88]. Moreover, due to a lack of critical eHealth literacy, older adults are more susceptible to the negative impact of misinformation on social media [44,51]. Therefore, establishing engaging and trustworthy social media health information channels tailored to older adults is imperative.

Currently, machine learning algorithms have been introduced into health management and monitoring for older adults, demonstrating positive effects in promoting self-management and preventing health risks [120,121]. For instance, the research by Zhao et al [122] used high-order hybrid clustering algorithms that integrated physical activity and medical perspectives, advancing personalized health care and rehabilitation for older adults. However, studies on optimizing social media algorithms for health information targeted at older adults remain relatively limited. Future research could enhance social media algorithms by prioritizing accurate, clear, and relevant health content tailored to older adults. Personalized recommendations could encourage older users to use reliable medical resources and participate in health promotion activities, thereby reducing their exposure to misinformation.

In addition, older adults are considered more inclined to trust health information disseminated through authoritative channels [81]. While social media provides an abundance of health information and convenience for older adults, the prevalence of misinformation often undermines their judgment. Therefore, it is crucial to encourage authoritative health care institutions to play a more active role in creating health content on social media. These institutions should regularly disseminate reliable health information through social media platforms while leveraging the social features of these platforms to offer knowledge-based question and answer sessions and interactive opportunities, such as expert online consultations.

Furthermore, the study by Nassetta and Gross [123] found that labels on social media can help mitigate the impact of misinformation, but this requires users to pay attention to the source of information. Thus, governments and health care institutions could collaborate to introduce reliable information verification mechanisms on social media platforms. Examples include implementing “trusted source” labels or certification marks for health information verified by authoritative organizations. These labels should be prominently displayed to enhance older adults’ trust in the credibility of information sources.

#### Integrating Social Support Systems to Foster Lifelong eHealth Learning for Older Adults

The findings of this review indicate that the potential of social media to enhance older adults’ eHealth literacy primarily lies in the social support it provides. First, social support serves as an important supplementary channel for older adults to access health resources. In this review, health information behaviors emerged as the most frequently discussed theme influencing older adults’ eHealth literacy. In addition to the health information directly obtained by older adults from social media, many health resources are accessed through the online social networks maintained by social media platforms [14,81].

Second, the social support older adults gain from the interactive advantages of social media can promote their health-sharing and communication behaviors, which have a significant positive impact on their eHealth literacy [80]. Moreover, as a crucial platform for online, group-based health interventions, the greatest advantage of social media lies in leveraging social support to encourage older adults to consistently use digital health technologies [83,85].

Although social support through social media has been found to have a positive effect on older adults’ mental health, health management, and health education in previous studies [15,124,125], it is still controversial whether social support is directly related to older adults’ eHealth literacy. Arcury et al [93] found no direct association between social support and eHealth literacy, whereas Kim and Sung [126] found that peer support was significantly related to the eHealth literacy of older individuals. This review did not find a direct association between the 2 but observed that social support provided by social media has the potential to promote the continued use of eHealth devices among older adults; however, this relationship has not been rigorously confirmed in the included literature [14,78,80,81,83,85]. Moreover, studies do find that peer support increases internet use among older adults [98]. Thus, the correlation between social support provided by social media and eHealth literacy among older adults may have been moderated through ongoing eHealth device use behaviors, and the relationship between the 3 could be explored in depth in the future.

While the direct relationship between social media and older adults’ eHealth literacy remains unclear, social media does reshape older adults’ health behaviors by linking their social networks and social support on online platforms. Studies suggested that the perceived positive effects of social support on health outcomes are not only limited to face-to-face interactions but are also applicable in the social media context [127,128]. However, a relatively small portion of the studies in this review (6/16, 38%) focused on the positive impact of social support on eHealth literacy among older adults. Most studies (10/16, 63%) lacked an exploration of older adults’ health-related interactions on social media, such as peer-to-peer interactions and interactions with health care professionals. To effectively leverage social media in building a sustainable mechanism to enhance older adults’ eHealth literacy, further exploration of their interactive behaviors on social media is needed, with an emphasis on the critical role of social support in encouraging older adults to actively engage in eHealth practices.

To this end, communities or health care institutions could use social media to establish virtual support groups focusing on different health topics, where volunteers regularly share health knowledge and encourage older adults to exchange their health experiences. Previous studies have called on hospitals and clinics worldwide to develop social media–supported medical apps, establish support groups, and encourage users to actively engage in social interactions with peers and health care providers [72].

In addition to the social support offered by online social media, offline social support remains indispensable. Despite the increased use of digital technologies among older adults driven by IT and the COVID-19 pandemic, research indicates that they still prefer traditional face-to-face interactions [129]. The development of technology should not be detached from the temperature of human beings, and the enhancement of eHealth literacy among older adults requires the intervention of “real people.” For example, older adults who lack functional digital skills often rely heavily on family members for technical assistance and advice [129]. However, as the caregiver burden on families increases, it is essential to involve community resources to help older adults establish a long-term mechanism for learning and practicing eHealth technologies.

Communities could host social media health education workshops to teach older adults how to use social media for accessing health information, using telemedicine services, and identifying misinformation. In addition, the role of social work volunteers should be emphasized to provide ongoing online technical support and guidance for older adults. As older adults gain more positive experiences from using social media to participate in health practices, they will likely engage more actively in the eHealth trend. This engagement will, in turn, enhance their eHealth literacy, allowing them to refine their behaviors and ultimately achieve a long-term positive feedback loop between social media health practices and eHealth literacy.

### Limitations

This review has several limitations. First, we only searched for keywords in the abstracts and titles, which, on the one hand, led to a large number of studies that used social media to recruit respondents unrelated to the topic appearing in the first search results. On the other hand, it could have resulted in the omission of articles in the primary content of the text dealing with the correlation between social media and eHealth literacy among older individuals. Second, the sample size for this review was relatively small due to the specificity of the research topic. Third, the thematic analysis conducted in this review was qualitative, and some (6/16, 38%) of the included studies did not directly examine the relationship between social media and eHealth literacy. Instead, they provided indirect evidence or theoretical suggestions that social media might enhance eHealth literacy in older adults. Therefore, the conclusions drawn in this review are primarily based on correlation rather than causation. Finally, the scarcity of existing research investigating this specific correlation highlights the need for more empirical studies that directly explore how social media impacts older adults’ eHealth literacy. Future research should address these gaps by using diverse methodologies and larger sample sizes to establish more comprehensive and definitive conclusions.

### Conclusions

Given the significant potential of social media in enhancing eHealth literacy among older individuals and the need to improve eHealth literacy among this population, this review sought to investigate the correlation between the social media use habits of older adults and their eHealth literacy. The review analyzed studies conducted from January 2000 to May 2024. The results found that social media and older adults’ eHealth literacy influence and interact with each other. Older adults’ social media use behaviors, including social media use habits and health behaviors, have a significant impact on their eHealth literacy, suggesting that social media has significant potential to enhance eHealth literacy among older adults. In turn, older adults’ eHealth literacy affects their willingness to adopt digital technologies, such as social media, as well as their performance of social media behaviors for health purposes. Promoting a virtuous cycle of interaction between the 2 fosters active aging and helps establish a long-term mechanism to enhance eHealth literacy among older adults. Achieving this goal requires a comprehensive consideration of factors that influence older adults’ use of social media to improve eHealth literacy, including social media penetration, insufficient digital skills, misinformation on social media, age, privacy and safety, and cultural norms and values. On the basis of the results of the review, this study proposes several future considerations for improving eHealth literacy among older adults through social media.

## Appendix

Multimedia Appendix 1 PRISMA checklist.

Multimedia Appendix 2 Searching strategies.

Multimedia Appendix 3 Conceptual matrix for thematic analysis of social media behavior.

Multimedia Appendix 4 Quality assessment for the studies reviewed.

## Abbreviations

mHealth: mobile health
PRISMA: Preferred Reporting Items for Systematic Reviews and Meta-Analyses
QA: quality assessment
RQ: research question
SEM: social-ecological model
VHC: virtual health community

## Data Availability

All data generated or analyzed during this study are included in this published article and its supplementary information files.
