# Supplementary material for: Social Media and eHealth Literacy Among Older Adults: Systematic Literature Review
Source: J Med Internet Res. 2025 Mar 26;27:e66058. doi: 10.2196/66058 (PMC11982777; doi:10.2196/66058)
Supplement: Multimedia Appendix 2 [file jmir_v27i1e66058_app2.docx]

| **Appendix 2. Searching strategies** | | |
| --- | --- | --- |
| Scopus | TITLE-ABS-KEY(("e-health literacy" OR "eHealth literacy" OR "telehealth literacy" OR "mHealth literacy" OR "health literacy" OR "medic* literacy" OR "digital literacy" OR "information literacy" OR "media literacy" OR "internet literacy" OR "computer literacy" OR "virtual literacy" OR "intelligent literacy" OR "technolog* literacy" OR "web-based literacy" OR "web based literacy" OR "Web 2.0 literacy" OR "ICT literacy" OR "Information and Communication Technology literacy") AND (old* OR elder* OR senior* OR aging* OR ageing* OR aged* OR geriatric* OR gerontolog* OR retiree* OR "baby boomer" OR pensioner* OR "silver generation") AND ("social media" OR "social network*" OR "social platform*" OR "social web*" OR "web 2.0" OR "online social*" OR "digital social*" OR "virtual social*" OR "social channels" OR "social apps" OR "community media*" OR "community platform" OR Facebook OR "You Tube" OR WhatsApp OR Instagram OR WeChat OR "Tik Tok" OR Douyin OR Telegram OR Snapchat OR Kuaishou OR "Sina Weibo" OR QQ OR Twitter OR Pinterest)) | 840 |
| Web of science | TS=(("e-health literacy" OR "eHealth literacy" OR "telehealth literacy" OR "mHealth literacy" OR "health literacy" OR "medic* literacy" OR "digital literacy" OR "information literacy" OR "media literacy" OR "internet literacy" OR "computer literacy" OR "virtual literacy" OR "intelligent literacy" OR "technolog* literacy" OR "web-based literacy" OR "web based literacy" OR "Web 2.0 literacy" OR "ICT literacy" OR "Information and Communication Technology literacy") AND (old* OR elder* OR senior* OR aging* OR ageing* OR aged* OR geriatric* OR gerontolog* OR retiree* OR "baby boomer" OR pensioner* OR "silver generation") AND ("social media" OR "social network*" OR "social platform*" OR "social web*" OR "web 2.0" OR "online social*" OR "digital social*" OR "virtual social*" OR "social channels" OR "social apps" OR "community media*" OR "community platform" OR Facebook OR "You Tube" OR WhatsApp OR Instagram OR WeChat OR "Tik Tok" OR Douyin OR Telegram OR Snapchat OR Kuaishou OR "Sina Weibo" OR QQ OR Twitter OR Pinterest)) | 501 |
| PubMed | (("old"[Title/Abstract] OR "older"[Title/Abstract] OR "elder"[Title/Abstract] OR "elderly"[Title/Abstract] OR "senior"[Title/Abstract] OR "aging"[Title/Abstract] OR "ageing"[Title/Abstract] OR "aged"[Title/Abstract] OR "gerontolog*"[Title/Abstract] OR "retiree*"[Title/Abstract] OR "baby boomer"[Title/Abstract] OR "pensioner*"[Title/Abstract] OR "silver generation"[Title/Abstract]) AND ("e-health literacy"[Title/Abstract] OR "eHealth literacy"[Title/Abstract] OR "telehealth literacy"[Title/Abstract] OR "mHealth literacy"[Title/Abstract] OR "health literacy"[Title/Abstract] OR "medic* literacy"[Title/Abstract] OR "digital literacy"[Title/Abstract] OR "information literacy"[Title/Abstract] OR "media literacy"[Title/Abstract] OR "internet literacy"[Title/Abstract] OR "computer literacy"[Title/Abstract] OR "virtual literacy" [Title/Abstract] OR "intelligent literacy"[Title/Abstract] OR "technolog* literacy"[Title/Abstract] OR "web-based literacy" [Title/Abstract] OR "web based literacy"[Title/Abstract] OR "Web 2.0 literacy" [Title/Abstract] OR "ICT literacy"[Title/Abstract] OR "Information and Communication Technology literacy"[Title/Abstract]) AND ("social media"[Title/Abstract] OR "social network*"[Title/Abstract] OR "social platform*"[Title/Abstract] OR "social web*"[Title/Abstract] OR "web 2.0"[Title/Abstract] OR "online social*"[Title/Abstract] OR "digital social*"[Title/Abstract] OR "virtual social*"[Title/Abstract] OR "social channels"[Title/Abstract] OR "social apps"[Title/Abstract] OR "community media*"[Title/Abstract] OR "community platform"[Title/Abstract] OR "Facebook"[Title/Abstract] OR "You Tube"[Title/Abstract] OR "WhatsApp"[Title/Abstract] OR "Instagram"[Title/Abstract] OR "WeChat"[Title/Abstract] OR "Tik Tok"[Title/Abstract] OR "Douyin"[Title/Abstract] OR "Telegram"[Title/Abstract] OR "Snapchat"[Title/Abstract] OR "Kuaishou"[Title/Abstract] OR "Sina Weibo"[Title/Abstract] OR "QQ"[Title/Abstract] OR "Twitter"[Title/Abstract] OR "Pinterest"[Title/Abstract])) | 250 |
