# Supplementary material for: Social Media and eHealth Literacy Among Older Adults: Systematic Literature Review
Source: J Med Internet Res. 2025 Mar 26;27:e66058. doi: 10.2196/66058 (PMC11982777; doi:10.2196/66058)
Supplement: Multimedia Appendix 3 [file jmir_v27i1e66058_app3.docx]

| **Study** | **Social media usage habits** | | **Health Information** | | | | | | **Health Intervention** | **Health Decision-Making** | | **Self-Management** | | | **Telemedicine** | |
| --- | --- | --- | --- | --- | --- | --- | --- | --- | --- | --- | --- | --- | --- | --- | --- | --- |
|  | Social media usage frequency | Social media platform preference | Health information acquisition | Health information sharing | Health knowledge learning | Health information evaluation | Health information trust | Frequency of health information use | Online group intervention | Medical decision-making | Solving general health issues | Medical management | Lifestyle management | Health prevention | Communicating with healthcare providers | Accessing healthcare services |
| Ye (2024) |  |  | **V** | **V** |  |  |  | **V** |  |  |  |  | **V** | **V** |  |  |
| Matchanova et al. (2023) |  |  |  | **V** |  | **V** |  |  |  |  |  |  |  |  |  |  |
| Oh et al. (2023) |  |  | **V** |  |  |  |  |  |  |  |  | **V** |  |  | **V** |  |
| Vitolo et al. (2023) | **V** |  |  |  |  |  |  |  |  |  |  |  |  |  |  |  |
| Jang et al. (2023) |  |  | **V** |  |  |  |  |  |  |  |  |  |  | **V** |  |  |
| Kachentawa et al. (2023) |  |  | **V** | **V** |  |  |  |  |  | **V** |  |  |  | **V** |  |  |
| Lee & Ryu. (2023) |  |  |  | **V** |  |  |  |  | **V** |  |  |  |  |  |  |  |
| Wang & Zhang. (2023) |  |  | **V** | **V** |  |  |  |  |  |  |  |  |  |  |  |  |
| Liu et al. (2022) |  |  | **V** |  |  | **V** |  |  |  |  |  |  | **V** | **V** |  | **V** |
| Chai (2022 |  |  | **V** |  |  | **V** |  |  |  |  |  |  | **V** |  |  |  |
| Tan et al. (2022) |  |  | **V** |  |  | **V** | **V** |  |  | **V** |  |  |  |  |  |  |
| Chen et al. (2021) |  |  |  |  |  |  |  |  | **V** |  | **V** |  | **V** | **V** |  |  |
| Wu and Yu (2021) |  | **V** |  |  |  |  |  |  |  |  |  | **V** |  | **V** |  | **V** |
| Ubolwan et al. (2020) | **V** |  |  |  |  |  |  |  |  |  |  |  |  |  |  |  |
| Shang & Zuo et al. (2020) |  |  |  |  | **V** |  |  |  |  |  |  |  |  |  |  |  |
| Tennant et al. (2015) | **V** |  | **V** |  |  | **V** |  |  |  |  |  |  |  |  |  |  |
