# Supplementary material for: Social Media and eHealth Literacy Among Older Adults: Systematic Literature Review
Source: J Med Internet Res. 2025 Mar 26;27:e66058. doi: 10.2196/66058 (PMC11982777; doi:10.2196/66058)
Supplement: Multimedia Appendix 4 [file jmir_v27i1e66058_app4.docx]

**Appendix4. Quality Assessment for Studies Reviewed**

**Questions**

QA1. Is the purpose of the study clearly stated?

QA2. Is the interest and usefulness of the work clearly presented?

QA3. Is the study methodology clearly established?

QA4. Are the concepts of the approach clearly defined?

QA5. Is the work compared and measured with other similar work?

QA6. Are the limitations of the work clearly mentioned?

**Answer options for each criterion**

Yes = 1 mark

Not completely = 0.5 mark

No = 0 mark

**Assessment method**

An article was deemed to be of sufficient quality if it achieved an overall score exceeding 3.0 points.

| **Author & Year** | **QA1** | **QA2** | **QA3** | **QA4** | **QA5** | **QA6** | **Overall Score** |
| --- | --- | --- | --- | --- | --- | --- | --- |
| Matchanova et al.（2023） | 1 | 0.5 | 1 | 0.5 | 0.5 | 0.5 | 4 |
| Ye (2024) | 1 | 0.5 | 1 | 0.5 | 0.5 | 0.5 | 4 |
| Oh et al. (2023) | 1 | 1 | 1 | 1 | 0.5 | 1 | 5.5 |
| Vitolo et al. (2023) | 1 | 0.5 | 1 | 0.5 | 0.5 | 0.5 | 4 |
| Jang et al. (2023) | 1 | 0.5 | 1 | 0.5 | 0.5 | 0.5 | 4 |
| Kachentawa et al.（2023） | 1 | 0.5 | 1 | 1 | 0.5 | 0.5 | 4.5 |
| Lee & Ryu. （2023） | 1 | 1 | 1 | 1 | 0.5 | 0.5 | 5 |
| Wang & Zhang. （2023） | 1 | 0.5 | 0.5 | 1 | 0.5 | 0.5 | 4 |
| Liu et al. (2022) | 1 | 0.5 | 0.5 | 0.5 | 0.5 | 0.5 | 3.5 |
| Chai (2022) | 1 | 1 | 0.5 | 1 | 0.5 | 1 | 5 |
| Tan et al. (2022) | 1 | 0.5 | 1 | 0.5 | 0.5 | 1 | 4.5 |
| Chen et al. (2021) | 1 | 1 | 1 | 0.5 | 0.5 | 0.5 | 4.5 |
| Wu and Yu (2021) | 1 | 1 | 0.5 | 1 | 0.5 | 0.5 | 4.5 |
| Ubolwan et al. (2020) | 1 | 0.5 | 1 | 0.5 | 0.5 | 0.5 | 4 |
| Shang &Zuo et al. (2020) | 1 | 1 | 0.5 | 1 | 0.5 | 0.5 | 4.5 |
| Tennant et al. (2015) | 1 | 1 | 1 | 1 | 0.5 | 1 | 5.5 |
